# Novel function of a putative *MOC1* ortholog associated with spikelet number per spike in common wheat

**DOI:** 10.1038/srep12211

**Published:** 2015-07-22

**Authors:** Bin Zhang, Xia Liu, Weina Xu, Jianzhong Chang, Ang Li, Xinguo Mao, Xueyong Zhang, Ruilian Jing

**Affiliations:** 1 National Key Facility for Crop Gene Resources and Genetic Improvement/Institute of Crop Science, Chinese Academy of Agricultural Sciences, Beijing 100081, China

## Abstract

Axillary meristems play an important role in determining final plant architecture and floral structures. Tomato *Ls*, Arabidopsis *LAS* and rice *MOC1* are orthologous genes regulating axillary meristem initiation and outgrowth. Their functions are generally conserved but the functional specificities are divergent among species. Obvious differences between rice panicles and wheat spikes suggest the divergent functions of *MOC1* and its wheat ortholog. We show that *TaMOC1* might be involved in wheat spikelet development. TaMOC1 is a typical nucleus localized protein with transcriptional activation abilities. The variable N-termini of TaMOC1 protein is necessary for transcriptional activation. *TaMOC1* is highly expressed in ears with length of 2, 3 and 6 cm. Significant associations between the *TaMOC1-7A* haplotype and spikelet number per spike were observed in ten environments over 3 years and 2 sites. *TaMOC1-7A Hap*H, a favored haplotype acquired during wheat polyploidization, may make a positive contribution to spikelet number per spike. Based on evolutionary analysis, geographic distribution and frequency changes, *TaMOC1-7A Hap*H might be associated with wheat domestication and Chinese wheat breeding history. The pyramiding favorable alleles of *TaMOC1-7A Hap*H and *TaSnRK2.10* (*C*, associated with higher TGW) can improve both spikelet number per spike and TGW simultaneously.

A plant body plan is established in seed plants when a primary shoot apical meristem (SAM) and a root apical meristem are formed at embryogenesis^1^. During postembryonic growth, plants generate a multitude of growth axes by forming new meristems called axillary meristems (AMs). The AMs produce a few leaf primordia and grow to become axillary buds, which produce additional shoot systems and lateral branches^2^,^3^. After transition from vegetative to reproductive development, the SAM is transformed into an inflorescence meristem that differentiates into an inflorescence. Each inflorescence meristem initiates several AMs that subsequently develop into inflorescence branches^4^,^5^. Thus, AM plays an important role in determining final plant architecture and reproduction, which are of special significance in crop production. Grain yield in common wheat (*Triticum aestivum*) can be dissected into various components, including number of spikes per unit area, spikelet number per spike, number of fertile florets per spikelet, and grain weight^6^. Among them, tillers and spikelets are produced by AMs.

Genes and mutants that regulate AM initiation and outgrowth have been described in various plant species, such as *Blind*^7^ and *Lateral suppressor* (*Ls*)^8^ genes in tomato; *LATERAL SUPPRESSOR (LAS)*^9^ and *REGULATORS OF AXILLARY MERISTEMS (RAX)*^10^ genes in Arabidopsis; *barren stalk1* (*ba1*)^11^, *branched silkless1*(*bd1*)^12^, *indeterminate spikelet1* (*ids1*)^13^, *teosinte branched1* (*tb1*)^14^ genes and *barren inflorescence2* (*bif2*) mutant^15^ in maize; *MONOCULM 1* (*MOC1*)^16^, *MULTI-FLORET SPIKELET1* (*MFS1*)^17^, *OsMADS34*^18^, *TONGARI-BOUSHI1* (*TOB1*)^19^, *LAX1* and *FRIZZY PANICLE 2* (*FZP2*)^20^ genes in rice; *MORE SPIKELETS1* (*MOS1*)^21^ gene in brachypodium; *HvTB1*^22^ gene in barley. *MOC1* in rice is an ortholog of the tomato *Ls* and Arabidopsis *LAS* genes^16^. The *Ls*/*LAS*/*MOC1* genes belong to the plant-specific GRAS family whose proteins are proposed to function as transcription factors, and regulate various aspects of plant growth and development^23^.

In common wheat, reduced tillering in the *tiller inhibition* (*tin*) mutant is due to early cessation of tiller bud outgrowth during transition from the vegetative to reproductive stage^24^. The *tin* gene was mapped to the short arm of chromosome 1A^25^. In diploid wheat (*Triticum monococcum* subsp. *monococcum*), *tiller inhibition* (*tin3*) mutant with almost no tillering ability produces only one main culm and *tin3* gene is located on the long arm of chromosome 3A^m^ 26,27. The *tin* and *tin3* genes have not been cloned and isolated. Recently, wheat *Photoperiod-1* (*Ppd-1*), was reported to have a major inhibitory effect on paired spikelet formation by regulating the expression of *FLOWERING LOCUS T* (*FT*)^28^. In addition, wheat *FRIZZY PANICLE* (*FZP*) gene can drive supernumerary spikelets^29^. In this study, we report the isolation and characterization of *TaMOC1*, the wheat ortholog of rice *MOC1*. Results of haplotype analysis showed that *TaMOC1* was significantly associated with spikelet number per spike. Haplotype differences in *TaMOC1* varied with time of cultivar release and geographic distribution across ecological zones.

## Results

### Cloning and sequence analysis of *TaMOC1*

**Figure 1:**
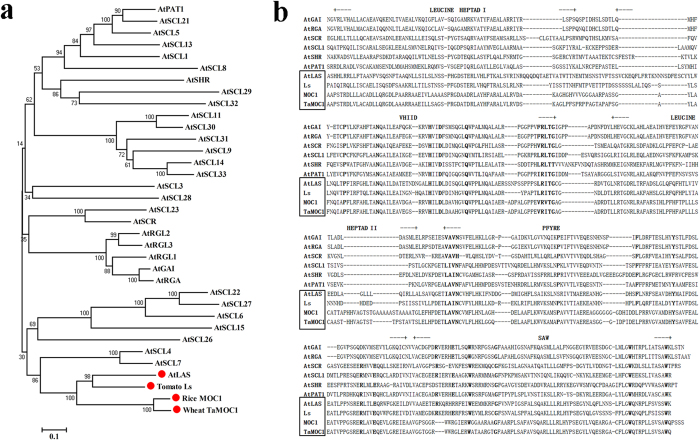
TaMOC1 belongs to the plant-specific GRAS family. (**a**) Phylogenetic analysis of GRAS proteins, including 32 members of *Arabidopsis thaliana* (At), tomato LS, rice MOC1, and wheat TaMOC1. The tree was constructed by the neighbor-joining method with 1,000 bootstrap replicates. Species and protein names are indicated at the end of each branch; and TaMOC1 and orthologous genes are marked with red dots. **(b)** Protein sequence alignment of TaMOC1, and orthologous tomato Ls, rice MOC1, Arabidopsis LAS, and other Arabidopsis GRAS gene products. The five recognizable motifs (LEUCINE HEPTAD I, VHIID, LEUCINE HEPTAD II, PFYRE and SAW) are indicated. Completely conserved residues within the VHIID, PFYRE and SAW motifs are highlighted in bold. Orthologs of TaMOC1 are included in the black rectangle.

*TaMOC1* cDNA contains a 1,290 bp ORF, and was predicted to encode 429 amino acid residues with a molecular mass of ~45.35 kDa. The TaMOC1 protein possesses a GRAS domain identified by a Pfam search and shares the highest sequence identity with rice MOC1 (82.6%), followed by 45.0 and 43.0% with the tomato Ls and Arabidopsis LAS proteins, respectively^8^,^9^. A neighbor-joining phylogenetic tree was constructed by alligning the full-length protein sequences with Clustal W (Fig. 1a). *TaMOC1* and its orthologous genes *AtLAS*/*LS*/*MOC1* were included into the same clade involved in axillary meristem development. In addition, the genes in the monocotyledons (*TaMOC1* in wheat and *MOC1* in rice) and those in the dicotyledons (*LS* in tomato and *AtLAS* in Arabidopsis) were distinguished in the phylogenetic tree as sub-clades. GRAS proteins share variable N-termini and highly conserved C-termini that can be divided by five motifs, viz. leucine heptad I, VHIID motif, leucine heptad II, PFYRE motif and SAW motif^23^. TaMOC1 has conserved characteristics of the five sequence motifs, and also contains completely conserved residues, such as P-N-H-D-Q-L in the VHIID motif (Fig. 1b).

### Subcelluar localization and transcription activity of TaMOC1 protein

**Figure 2:**
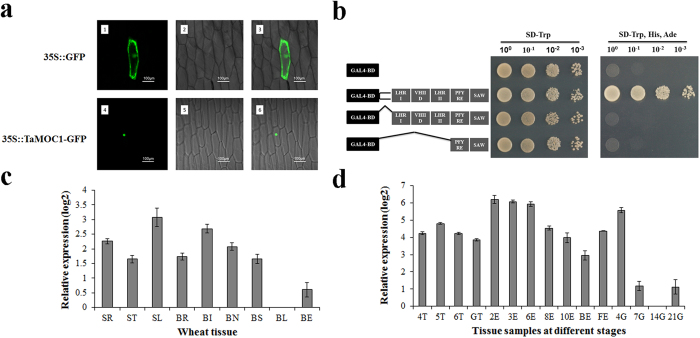
Subcellular localization, transactivation analysis and expression patterns of TaMOC1. (**a**) Subcellular localization of TaMOC1 in onion epidermal cells. The vector control (35S::GFP) and fusion proteins (35S::TaMOC1–GFP) were introduced into onion epidermal cells and observed with a laser scanning confocal microscope. Images are in dark field (1, 4), bright field (2, 5), and combined (3, 6). (**b**) Transcriptional activation activity of TaMOC1 in a modified yeast two-hybrid assay. The full-length ORF of *TaMOC1* and DNA fragments for different N-terminal truncations were introduced into the pGBKT7 vector. GAL4-BD represents GAL4 DNA binding domain. LHR I, VHIID, LHR II, PFYRE and SAW are the five motifs of GRAS domain in C-termini of TaMOC1. The broken lines indicate the truncated deletions. The empty pGBKT7 vector is the negative control. (**c**) Expression patterns of *TaMOC1* in different wheat tissues. SR, ST, and SL: roots, tillers buds, and leaf blades at the seedling stage; BR, BI, BN, BS, BL, and BE: roots, internodes, nodes, leaf sheaths, leaf blades, and ears at late booting. (**d**) Expression patterns of wheat *TaMOC1* during development of tiller buds, ears, and grain. 4T, 5T, 6T, and GT: tiller buds at the 4-leaf, 5-leaf, 6-leaf, and greening stages; 2E, 3E, 6E, 8E, and 10E: ears lengths of 2–10 cm; BE and FE: ears at the late booting and flowering stages; 4G, 7G, 14G, and 21G: grains at 4, 7, 14, and 21 days post-flowering. Actin was used as an internal control. The qRT-PCR results were reported using a log scale (log2). Bar: 2 × SE.

GRAS proteins are putative transcription factors. Prediction of subcellular localization using ProtComp Version 9.0 software suggested that TaMOC1 was a typical nuclear localized protein. To address this point, the recombinant construct of the TaMOC1-GFP fusion plasmid was transiently expressed in living onion epidermal cells by particle bombardment. The control GFP signal was detected in the whole onion epidermal cell. In contrast, TaMOC1-GFP was exclusively localized in the cell nucleus (Fig. 2a).

In order to evaluate the function of the TaMOC1 as a transcription factor, transcriptional activation experiments by a modified yeast two-hybrid assay were conducted. The GAL4-BD/TaMOC1 fusion proteins activated transcription in yeast, whereas the negative control (GAL4-BD alone) failed to do so (Fig. 2b). In addition, the fragment containing the whole GRAS domain and the one containing the PFYRE and SAW motifs of the GRAS domain in TaMOC1 had no transactivation ability in yeast. Deletion analyses suggested that the variable N-termini of the TaMOC1 protein was necessary for transcriptional activation.

### Expression pattern of *TaMOC1* in wheat

Quantitative real-time PCR were used to analyze the expression patterns of *TaMOC1*. *TaMOC1* was constitutively expressed in wheat, and mainly expressed in roots (SR), tiller buds (ST), and leaf blades (SL) at the seedling stage, in roots (BR), internodes (BI), nodes (BN), and leaf sheaths (BS) at the late booting stage (Fig. 2c). In order to investigate the expression pattern of *TaMOC1* in development of lateral branches at the vegetative (tillering) and reproductive (spikelet differentiation) stages, various wheat tissues at 15 developmental stages, including tiller buds, ears and grains were collected from the 4-leaf stage to 21 days post-flowering (Fig. 2d). *TaMOC1* was highly expressed in 2E, 3E, 6E and 4G, and only barely detected at 7G, 14G and 21G.

### Sequence polymorphism assays and genetic mapping

**Figure 3:**
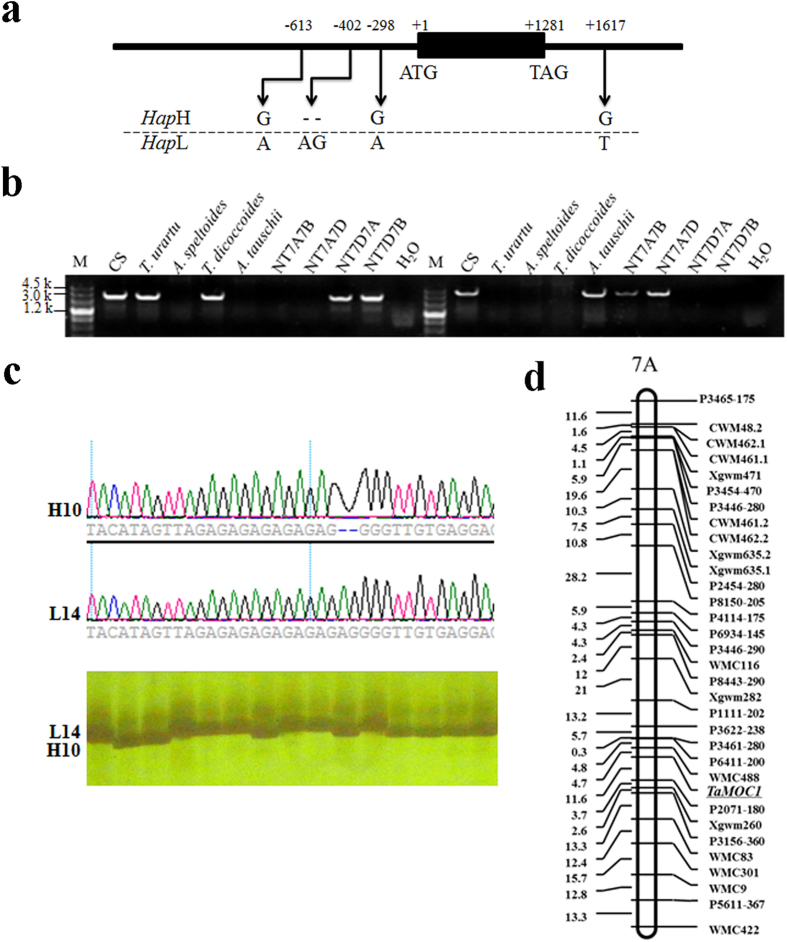
Nucleotide polymorphisms and genetic mapping of *TaMOC1-A*. (**a**) Nucleotide polymorphisms in two haplotypes identified in *TaMOC1-A* among 37 common wheat accessions. The polymorphic site and relative positions are indicated. “—” represents the deletion. (**b**) Location of *TaMOC1-A* and *TaMOC1-D* in chromosomes 7A (left) and 7D (right) using CS nullisomic-tetrasomic lines. Location in a particular chromosome is indicated by absence of expression when that chromosome is missing. M, Marker III (TransGen, Beijing, China). (**c**) A functional marker was developed based on the polymorphic indel (—/AG) site. (**d**) Linkage map of *TaMOC1-A* on wheat chromosome 7A based on SSR data from the Hanxuan 10 × Lumai 14 DH population.

The 5’ flanking regions of *TaMOC1* were obtained by blastn searches of the draft genome databases of the wheat A and D genome progenitors^30^,^31^. Genome-specific primer pairs for the A (TaMAF/TaMAR) and D (TaMDF/TaMDR) orthologs were designed from sequence differences. The fragments were about 2,700 and 4,200 bp in size. Four variants in the entire length of *TaMOC1-A* were detected among 37 accessions. Two haplotypes were formed by two SNPs (G/A, G/A) and one InDel (—/AG) in the 5’ flanking region (816 bp), and one SNP (G/T) in the 3’ flanking region (622 bp, Fig. 3a). The indel (—/AG) polymorphic site was chosen to develop a pair of functional markers TaMAMF/TaMAMR, designed specifically for the region flanking the indel (—/AG) (Fig. 3c). The Chinese wheat cultivars Hanxuan 10 and Lumai 14, parents of the DH population, possessed different haplotypes; the Hanxuan 10 allele was named *Hap*H and the Lumai 14 allele, *Hap*L (Fig. 3a).

Using Chinese Spring (CS) nullisomic-tetrasomic lines, *TaMOC1-A* and *TaMOC1-D* were located on homoeologous group 7 chromosomes (Fig. 3b). Target fragments produced by the A genome-specific primers (TaMAF/TaMAR) were detected only in accessions with chromosome 7A (CS, *T. urartu*, *T. dicoccoides*, NT7D7A and NT7D7B). Likewise, target fragments produced by the D genome-specific primers (TaMDF/TaMDR) were detected only in accessions with chromosome 7D (CS, *A. tauschii*, NT7A7B, NT7A7D). Using the DH population, *TaMOC1-A* was mapped to a region flanked by *WMC488* (4.7 cM) and *P2071-180* (11.6 cM) on chromosome 7A (Fig. 3d).

### Association of *TaMOC1-7A* haplotypes with spikelet number per spike

**Figure 4:**
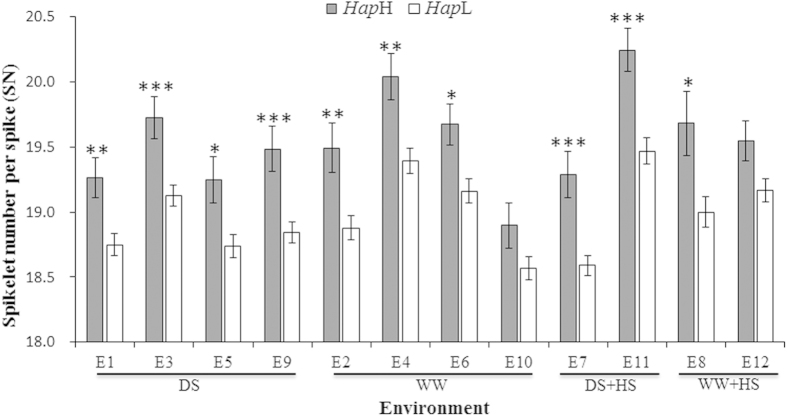
Contributions of *TaMOC1-7A Hap*H to higher spikelet number per spike in twelve environments. See caption to Table 1 for description of environments. *, **, *** Significant at *P* = 0.05, 0.01 and 0.001, respectively.

According to the analysis of population structure by 209 whole-genome SSR markers, Population 1 (262 accessions) consisted of two sub-populations, comprising 126 and 136 accessions^32^. An association analysis between *TaMOC1-7A* haplotypes and five agronomic traits was conducted using a general linear model (GLM), which accounted for population structure (Q) (Table 1). In twelve environments, no significant associations of *TaMOC1-7A* haplotypes with spike length (SL) and 1000-grain weight (TGW) were detected. There were only one and three instances of association with plant height (PH; E6) and grain number per spike (GN; E4, E5 and E11), respectively. However, significant associations between *TaMOC1-7A* haplotypes and spikelet number per spike (SN) were observed in 10 environments over 3 years and 2 sites (Table 1). The phenotypic variation for spikelet number per spike explained by *TaMOC1-7A* haplotypes ranged from 2.57% in E6 to 6.00% in E7. In particular, *Hap*H was significantly associated with higher spikelet number per spike in almost all environments except for E10 and E12 (Table 1 and Fig. 4). Thus, *TaMOC1-7A Hap*H may represent a favorable haplotype that could make a positive contribution to spikelet number per spike.

### Possible selection for *TaMOC1-7A Hap*H along with spikelet number per spike during the history of wheat breeding

**Figure 5:**
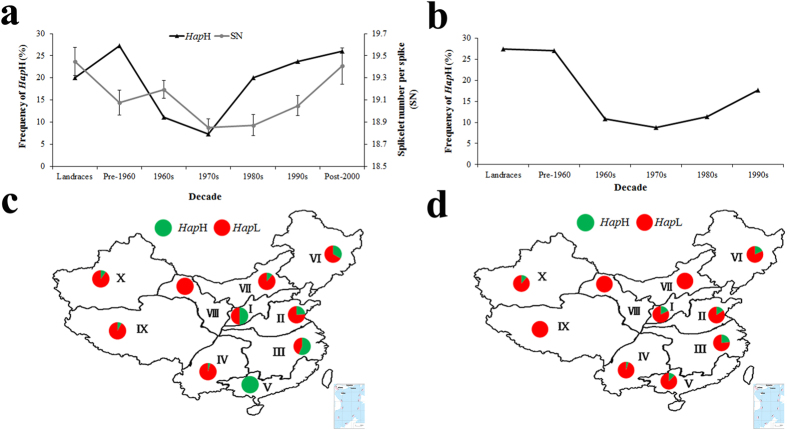
Frequency and geographic distribution of *TaMOC1-7A Hap*H in Chinese wheat landraces and modern cultivars over different decades and across the ten Chinese wheat ecological production zones. (**a**) Frequencies of *Hap*H and changes of spikelet number per spike in Population 1 (262 accessions) grown in 12 environments. Ten landraces are represented. Eleven, 27, 55, 40, 59 and 50 accessions were released in the pre-1960s, 1960s, 1970s, 1980s, 1990s and post-2000, respectively. Ten accessions with unknown release dates were excluded. Bars indicate 2 × SE. (**b**) Verification of frequency distributions of *Hap*H in Populations 2 (157 landraces) and 3 (348 modern cultivars). There were 37, 55, 102, 106 and 34 accessions released in the pre-1960s, 1960s, 1970s, 1980s and 1990s, respectively. Fourteen accessions with unknown release dates were excluded. (**c**) Distribution of *TaMOC1-7A* haplotypes in 157 landraces. (**d**) Distribution of *TaMOC1-7A* haplotypes in 348 modern cultivars. I, Northern Winter Wheat Zone; II, Yellow and Huai River Valleys Facultative Wheat Zone; III, Middle and Lower Yangtze Valleys Autumn-Sown Spring Wheat Zone; IV, Southwestern Autumn-Sown Spring Wheat Zone; V, Southern Autumn-Sown Spring Wheat Zone; VI, Northeastern Spring Wheat Zone; VII, Northern Spring Wheat Zone; VIII, Northwestern Spring Wheat Zone; IX, Qinghai-Tibetan Plateau Spring-Winter Wheat Zone; X, Xinjiang Winter-Spring Wheat Zone. The maps were generated using Mapinfo Professional software.

Data for Population 1 (262 accessions) grown in 12 environments suggests that spikelet number per spike decreased with the change from landraces to modern cultivars released in the 1970s, but then increased in modern cultivars released after the 1970s (Fig. 5a). The trend of change in frequency of *Hap*H over decades was relatively constant as verified in Populations 1, 2 (157 landraces) and 3 (348 modern cultivars) (Population 2 and 3 were from the Chinese wheat mini-core or core collection; Fig. 5a,b). In Population 1, *Hap*H frequencies in landraces and modern cultivars released pre-1960 were 20% and 27%; and gradually decreased to 7.27% in 1970s modern cultivars; then gradually increased to 26% after 2000. In Populations 2 and 3, *Hap*H frequencies exhibited the same trends with early decreases and subsequent increases; the frequencies were 27.45, 27.03, 8.82 and 17.65% in landraces and modern cultivars from pre-1960, and modern cultivars from the 1970s and 1990s, respectively. These results suggest that *TaMOC1-A* might have been under selection during breeding.

### Geographic distribution of *TaMOC1-7A* haplotypes in ten Chinese wheat production zones

Common wheat is sown in both autumn and spring. According to their sowing period and requirement for vernalization, wheat is classified into three types in China, VIZ. spring, facultative and winter wheat. The Chinese wheat production area is divided into ten major agro-ecological production zones based on ecological conditions, variety type and growing season^33^,^34^. Winter-habit and facultative wheat (autumn-planted) is grown in Zone I and Zone II; autumn-planted spring wheat is grown in Zone III, Zone IV and Zone V; spring-planted spring wheat is grown in Zone VI, Zone VII and Zone VIII; spring-planted spring wheat and autumn-planted winter wheat in ecotones Zone IX and Zone X^34^. The geographic distributions of *TaMOC1-7A* haplotypes were evaluated by 157 landraces and 348 modern cultivars covering all zones (Fig. 5c,d). Similar trends were observed in landraces and modern cultivars; *TaMOC1-7A Hap*H mainly occurred in the autumn-sown wheat zones (I, II, III, V), especially in landraces. In the autumn-sown wheat zones *Hap*H was most frequent in the Middle and Lower Yangtze Valleys Autumn-Sown Spring Wheat Zone (III; 56.52% among landraces; 23.53% among modern cultivars) and Southern Autumn-Sown Spring Wheat Zone (V; 100% among landraces). In spring wheat zones the frequency of *Hap*H was higher in the Northeastern Spring Wheat Zone (VI; 33.33% among landraces; 18.18% among modern cultivars) than the Northern Spring (VII) and Northwestern Spring (VIII) Wheat Zones. This suggested that *Hap*H was mainly used in lower latitude areas of the autumn-sown wheat zones and higher latitude areas of the spring wheat zones.

### Improving both spikelet number per spike and 1000-grain weight by combining favorable alleles of *TaMOC1-7A* and *TaSnRK2.10*

Sucrose non-fermenting1-related protein kinase (SnRK) is a widely occurring Ser/Thr protein kinase in plants. SnRKs participate in transduction of various signaling pathways and can affect carbon metabolism to increase 1000-grain weight^35^. Earlier work in our laboratory demonstrated that a non-synonymous mutation from G to C in *TaSnRK2.10* introduced a superior allele for improvement of 1000-grain weight^36^. In this study, we further verified the effect of *TaSnRK2.10* on 1000-grain weight (Supplementary Fig. S1). There were significant differences in 1000-grain weight between *TaSnRK2.10-C* and *TaSnRK2.10-G* in 10 of the 12 envrironments (except E9 and E11). *TaSnRK2.10* had no effect on spikelet number per spike in all 12 environments (Supplementary Fig. S1).

To explore the effectiveness of improving both spikelet number per spike and 1000-grain weight, we analyzed the efficacy of combining favorable alleles of *TaMOC1-7A* (*Hap*H) and *TaSnRK2.10*-C in eight environments (E1-E8; Supplementary Table S1). There was no significant difference in spikelet number per spike between *TaMOC1-7A Hap*H and *Hap*L in E10 and E12; and no significant difference in 1000-grain weight between *TaSnRK2.10-C* and *TaSnRK2.10-G* in E9 and E11. Thus, E9-E12 were excluded from consideration. Based on eight environments, the average spikelet number per spike and 1000-grain weight of genotypes combining both favorable alleles were 19.0–19.9 and 35.3–44.4 g, respectively; those of genotypes without both favorable alleles were 18.5–19.3 and 32.7–40.9 g (Supplementary Table S1). Therefore, combining *Hap*H and *TaSnRK2.10-C* could improve both spikelet number per spike and 1000-grain weight simultaneously.

### Evolutionary analysis of *TaMOC1-A*

**Figure 6:**
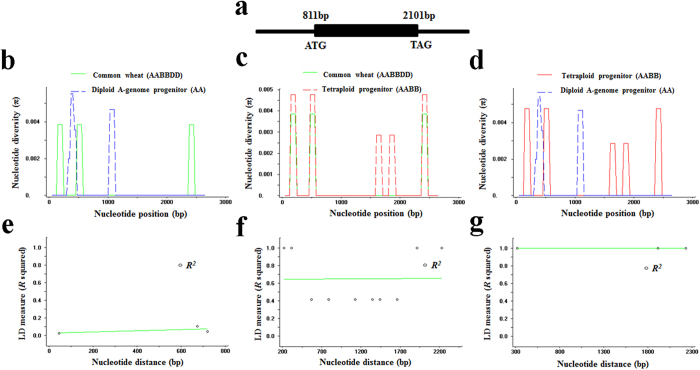
Nucleotide diversity (*π*) and composite plots of linkage disequilibria (LD) in *TaMOC1-A.* Structure of *TaMOC1* is shown in (**a**). Sliding-window analyses between common wheat (AABBDD) and diploid accessions (AA, **b**), between common wheat and tetraploid accessions (AABB, **c**), and between diploid A-genome progenitor and tetraploid progenitor accessions (**d**). *π* is shown in a sliding window of 100 bp using a step size of 25 bp. Composite plots of linkage disequilibria (LD) in *TaMOC1-A* in diploid (**e**), tetraploid (**f**), and common wheat (**g**) accessions. *R*^*2*^measures of LD are shown as functions of distance for all loci examined. LD values between all pairs of nucleotide variations are plotted. The Figure shows logarithmic trend lines.

To reveal whether *TaMOC1-A* might have been selected during wheat domestication, nucleotide and haplotype diversity were evaluated in a panel of 54 wheat germplasms, including 37 accessions of common wheat and 17 accessions of wild wheat-related species, viz. 10 A-genome and 7 AABB-genome accessions. The sequence structure of *TaMOC1* used for evolutionary analysis is shown in Fig. 6a. The nucleotide diversities (*π*) of the entire *TaMOC1-A* regions were 0.00038, 0.00074 and 0.00043 in diploid, tetraploid and common wheat, respectively. The higher value of *π* in the tetraploid progenitor than in common wheat suggested selection pressure during the second polyploidization. Sliding-window analysis showed nucleotide variants in the entire region of *TaMOC1-A* differed between diploid progenitor and common wheat and tetraploid progenitor (Fig. 6b,d); the tetraploid progenitors included the nucleotide variations and the two haplotypes identified in common wheat (Fig. 6c). Linkage disequilibria (LD) were estimated between all pairs of polymorphic sites in *TaMOC1-A* using the *R*^*2*^ statistic, and the significances of pairwise disequilibrium comparisons were assessed by Fisher’s exact test^37^. No significant LD was detected in *TaMOC1-A* in diploid A-genome progenitor accessions (Fig. 6e), whereas clear LD was observed in the tetraploid accessions (Fig. 6f); and complete LD was detected in common wheat (Fig. 6g). The population pairwise *F*_*st*_ values were significantly different between common wheat and diploid accessions (*F*_*st*_ = 0.4646, *P *< 0.0001), as well as between common wheat and tetraploid accessions (*F*_*st*_ = 0.5197, *P *< 0.0001). These results suggest that *TaMOC1-A* might be a domestication-related gene.

## Discussion

The functions of homologous genes across genomes and species are generally conserved, but functional specificity may be divergent. Tomato *Ls*^8^, Arabidopsis *LAS*^9^, rice *MOC1*^16^ and wheat *TaMOC1* are orthologous genes (Fig. 1). Tomato *Ls* and Arabidopsis *LAS* specifically regulate the formation of axillary meristems (AMs) during vegetative development. *ls* and *las* mutants are characterized by almost complete suppression of axillary shoots during vegetative development, but normal axillary shoot development can occur following transition to the reproductive phase. In addition, *ls* flowers fail to develop petals and display reduced male and female fertility, but such defects are not observed in the Arabidopsis *las* mutant^9^,^38^. Rice *MOC1* functions in the formation and outgrowth of tiller buds (AMs) during both the vegetative and reproductive phases^16^. Differences between *moc1* and *ls*/*las* mutants reflect fundamental variation between monocotyledonous tillering and dicotyledonous branching. Moreover, differences between rice panicles and wheat spikes may reflect functional divergence of *MOC1* and *TaMOC1*. Association analysis between *TaMOC1-7A* haplotypes and five agronomic traits (plant height, spike length, spikelet number per spike, grain number per spike, and 1000-grain weight) was conducted in wheat to reveal its function. In twelve environments, contrary to the negative effects of rice *MOC1* on plant height, only one instance (environment E6) of association of *TaMOC1-7A* haplotypes with plant height was detected, whereas robustly significant associations with spikelet number per spike were observed in ten environments over 3 years and 2 sites (Table 1). In addition, TaMOC1 is a typical cell nucleus localized protein with transcriptional activation motifs (Fig. 2a,b)^8^,^16^. The variable N-termini of TaMOC1 protein is necessary for transcriptional activation (Fig. 2b). It was reported that variability in the N-termini of GRAS proteins might mediate a number of different interactions involving the basic transcriptional machinery and accessory proteins^23^.

Wheat, which was domesticated about 10,000 years ago, has become one of the current major crops^39^. Domestication is accompanied by bottlenecks that leads to reduced genetic diversity^40^. However, wheat experienced two rounds of polyploidization, and compensated for such bottlenecks by capturing part of the genetic diversity of the progenitors and by generating new diversity by mutation in dynamic wheat genomes permitted by genetic duplication and redundancy^39^. By acquiring the D genome from *Ae. tauschii*, hexaploid wheat gained broader adaptability to a wide range of environments^30^. In addition, during the domestication process of crops, some essential genes underwent changes to new but related functions. For example, a deletion within the upstream region of *Ppd-D1* led to a widely distributed photoperiod-insensitive allele^39^. Compared to the diploid A-genome progenitor, the tetraploid progenitor developed novel alleles of *TaMOC1-A* reflected by: (1) higher nucleotide diversity (*π*) in the tetraploid progenitors species than in the A-genome progenitor, and (2) the tetraploid accessions included both haplotypes (*Hap*H and *Hap*L) of *TaMOC1-A* in common wheat whereas neither found in the diploid A-genome progenitor (Fig. 6a,d). Moreover, all other haplotypes except the two in common wheat were lost during subsequent polyploidization and domestication. Linkage disequilibrium (LD) represents nonrandom association between allelic polymorphisms at a locus. The gradual increasing LD in *TaMOC1-A* from no significant LD in diploid A-genome accessions to complete LD in common wheat suggests that selection occurred on *TaMOC1-A* (Fig. 6e,g). Pairwise *F*_*st*_ values among diploid A-genome, tetraploid and common wheat accessions also revealed that *TaMOC1-A* might be a domestication-related gene.

The spikelet is the basal unit of inflorescence in grasses and is crucial for reproductive success and final yield^41^. *TaMOC1-7A* may play a role in spikelet development in wheat (Table 1 and Fig. 2c,d). In addition, loci determining spikelet number per spike were reported to be located in the distal region of chromosome 7AL, the same region as the location of *TaMOC1-7A* (Fig. 3d)^42^. Because of positive selection during breeding, crop genetic diversity can be reduced by genetic bottlenecks. In addition, the frequencies of the most adapted alleles that meet human needs have increased in modern cultivars^43^. Similarly, in our study only *TaMOC1-7A Hap*H and *Hap*L were detected in common wheat (Fig. 6a,d). *Hap*H, a favorable haplotype, seems to make a positive contribution to spikelet number per spike (Fig. 4). According to breeding history over decades, spikelet number per spike underwent negative selection during the early breeding years, but was positively selected after 1970 (Fig. 5a). Moreover, spikelet number per spike is higher in varieties grown in autumn-sown wheat zones and is also higher in Northeastern Spring than Northern and Northwestern Spring Wheat Zones^33^. *TaMOC1-7A Hap*H coincides with the above wheat breeding history in China, indicating that it might have been selected along with spikelet number per spike (Fig. 5), further supporting the presumed value of *Hap*H in breeding programs over past decades.

In wheat, final yield (per m^2^) is considered as the product of [(PI m^−2^ *Sp PI^−1^ *sp Sp^−1^ *Gr sp^−1^) *IGWt], where PI m^−2^, Sp PI^−1^, sp Sp^−1^, Gr sp^−1^, IGWt stand for plants m^−2^, spikes plant^−1^, spikelets spike^−1^, grains spikelet^−1^, and average individual grain weight, respectively^44^. However, these yield components are almost negatively related to each other except the average individual grain weight, i.e., as one component increases, others will decrease. And the magnitude of the parameters of these relationships is also variable^44^,^45^,^46^. It has been reported that spikelet number per spike didn’t explain the differences in grain number per spike, and spikelet number per spike was closely associated with the grain number each spikelet could set^47^. Reynolds *et al.* even proposed a hypothesis, i.e., would it be sensible to try to select for a higher number of spikelet per spike if it brings about an associated reduction in number of grains per spikelet^44^? Thus, the complex relationships between those yield components may explain why there was only three and zero significant associations of *TaMOC1-7A* haplotypes with grain number per spike and 1000-grain weight in twelve environments, even though *TaMOC1-7A Hap*H was associated with a modest increase in spikelet number per spike in 10 environments over 3 years and 2 sites (Table 1 and Fig. 4). Significant differences in 1000-grain weight were detected between *TaSnRK2.10-C* and *TaSnRK2.10-G* in 10 of 12 envrironments, but *TaSnRK2.10* had no effect on spikelet number per spike in all 12 environments (Supplementary Fig. S1). Both spikelet number per spike and 1000-grain weight are important yield-related components in wheat. Gene pyramiding should be an effective way to achieve breeding progress by marker-assisted selection. In rice, yield and quality are typically negatively correlated with each other^48^. However, pyramiding elite alleles of *GS3* and *OsSPL16* underlying grain size and shape can be effectively used to simultaneously improve grain quality and yield^49^. In this study, we investigated the efficacy of combining favorable alleles of *TaMOC1-7A* (*Hap*H) and *TaSnRK2.10* (*C*, associated with higher TGW) in eight environments (Supplementary Table S1). The results suggest that pyramiding favorable alleles of *TaMOC1-7A Hap*H and *TaSnRK2.10-C* can improve both spikelet number per spike and TGW simultaneously.

## Materials and Methods

### Plant materials and measurement of agronomic traits

Common wheat cultivar Yanzhan 4110 was used for gene cloning and expression analysis. Thirty-seven cultivars (Supplementary Table S2) with wide variation in tiller number and spike-related traits were used for detecting sequence differences in *TaMOC1*. Thirty seven cultivars and 17 accessions of wheat relative species were chosen for evolutionary studies. The wild relative species consisted of 10 accessions of the diploid A-genome progenitor species *Triticum urartu* with the AA genome (UR1, UR102, UR200, UR201, UR202, UR204, UR205, UR206, UR207 and UR208) and 7 accessions of the tetraploid progenitor *T. dicoccoides* with the AABB genome (DS1, DS6, DS8, PS5, P09, DM50 and DM51). A set of Chinese Spring (CS) nullisomic-tetrasomic lines was used for chromosome location. A doubled haploid (DH) population derived from the cross Hanxuan 10 × Lumai 14 was used for fine mapping.

Three populations of hexaploid winter wheat were also used. Population 1 (262 accessions, Supplementary Table S3) was used for association analysis; 254 accessions were from China, three from the USA, two from Australia, two from Italy, and one from Romania, and included 209 modern varieties, 43 advanced lines and 10 landraces^50^. The cultivars from China were mainly from the Northern Winter Wheat and Yellow and Huai River Valleys Facultative Wheat Zones. Population 2 (157 landraces, Supplementary Table S4) and Population 3 (348 modern cultivars, Supplementary Table S5) were used for temporal haplotype and geographic distribution analyses. Population 2 mainly came from the Chinese wheat mini-core collection (MCC), which represents more than 70% of the genetic diversity of the full Chinese germplasm collection, and Population 3 was from the Chinese wheat core collection (CC)^51^,^52^.

Population 1 was planted at Changping (116°13´E; 40°13´N) and Shunyi (116°56´E; 40°23´N), Beijing, over 3 years for measuring agronomic traits, viz., plant height (PH), spike length (SL), spikelet number per spike (SN), grain number per spike (GN), and 1000-grain weight (TGW). Two water regimes, rain-fed (drought stressed, DS) and well-watered (WW), were applied at each site. The plantings were in 2009 at Changping, 2010 at Changping and Shunyi, and 2011 at Shunyi. The rainfalls during the growing seasons were 192 mm, 131 mm and 180 mm, respectively. The WW plots were irrigated with 750 m^3^/ha (75 mm) at each of the pre-overwintering, booting, flowering and grain filling stages. In addition, a controlled experiment was conducted at Shunyi; polythene covers were placed over the plots at heading to increase the temperature, and thereby simulate heat stress (HS). E1 to E12 indicate the environments at Changping in 2009 under DS and WW, Changping in 2010 under DS and WW, Shunyi in 2010 under DS, WW, DS + HS, and WW + HS, and Shunyi in 2011 under DS, WW, DS + HS and WW+HS, respectively.

### Cloning *TaMOC1*

In order to obtain the coding and flanking regions sequences of *TaMOC1*, the cDNA sequence of rice *MOC1* [GenBank: AY242058.1] was used for a blast search against the CerealsDB full-length cDNA library of the wheat D-genome progenitor *Aegilops tauschii*, and the draft genome databases of the wheat A (*Triticum urartu*) and D genome progenitors^30^,^31^,^53^. Three pairs of primers were designed according the assembled *TaMOC1* putative sequence, viz. (1) full-length cDNA of *TaMOC1* amplified using TaMF and TaMR (5’-CTATTAAACCCCCCCAGAGA-3’ and 5’-GCATGCACAGACCACAGAGT-3’), (2) the A genome-specific primer pair TaMAF and TaMAR (5’-GTGGTAAGATATGATAGATGCAAGT-3’ and 5’-GCAAAAAAGTTCATGATGCA-3’) designed to amplify *TaMOC1-A*, including the 5’ and 3’ flanking regions, and (3) the D genome-specific primer pair TaMDF and TaMDR (5’-CTGGATCTACCGACTGATACG-3’ and 5’-GCCGTGCAAGTGAGCTATA-3’) used to amplify *TaMOC1-D* including the 5’ and 3’ flanking regions. The cDNA and genomic DNA of common wheat cultivar Yanzhan 4110 were used as templates. The gene structure of *TaMOC1* was determined using DNASTAR Lasergene 7.1.0 (DNASTAR, Inc., Madison, WI, USA) through alignment of the amplified cDNA and genomic DNA sequences.

### Phylogenetic analysis

Excluding a pseudogene (*AtSCL16*), 32 members of the *Arabidopsis thaliana* GRAS family were downloaded from TAIR (http://www.arabidopsis.org/index.jsp). Rice *MOC1* [AY242058.1] and tomato *Ls* [NM_001247250.1] homologs of *TaMOC1* were obtained from NCBI (http://www.ncbi.nlm.nih.gov/). By comparing full-length protein sequences aligned with the Clustal W algorithm within MEGA 4.1, a neighbor-joining phylogenetic tree was constructed based on 1,000 bootstrap replicates^54^.

### Subcellular localization of TaMOC1 protein

The full-length ORF of *TaMOC1* was fused upstream of the GFP gene in the pJIT163-GFP expression vector under control of the CaMV35S promoter. The primers with restriction sites used for GFP fusion subcloning were 5’-AGCGAAGCTTATGATCGGCTCACTCCACTCT-3’ (*Hin*dIII site underlined) and 5’-ACTCGGATCCCTGCCACGCCGACACG-3’ (*Bam*HI site underlined). The 35S::TaMOC1-GFP fusion construct and GFP as control were transformed into onion epidermal cells by biolistic bombardment (Helios; Bio-Rad, Richmond, CA, USA). After incubation on Murashige and Skoog medium at 28 °C for 36–48 h, the transformed cells were observed with a laser scanning confocal microscope (Leika TCS‐NT, Heidelberg, Germany).

### Transactivation activity assay

*Saccharomyces cerevisiae* strain AH109 and GAL4-based Matchmaker Two-Hybrid System (Clontech, Palo Alto, CA, USA) were used in a transactivation activity assay. The full-length ORF of *TaMOC1* and N-terminal truncation versions were amplified, and inserted into pGBKT7 to produce in-frame fusions to the GAL4-binding domain. The *TaMOC1* coding region was amplified using primers 5’-AGCTGAATTCATGATCGGCTCACTCCACTCTTC-3’ (*Eco*RI site underlined) and 5’-ACTCGGATCCCCTACTGCCACGCCGACAC-3’ (*Bam*HI site underlined). Two pairs of primers, 5’-AATTGAATTCATGACGCGGGACCTCGTGCT-3’ (*Eco*RI site underlined) and 5’-ACTCGGATCCCCTACTGCCACGCCGACAC-3’ (*Bam*HI site underlined), and 5’-AATTGAATTCATGCTGGCCGTGAACTGCGT-3’ (*Eco*RI site underlined) and 5’-ACTCGGATCCCCTACTGCCACGCCGACAC-3’ (*Bam*HI site underlined), were used to amplify two N-terminal truncation versions, the former containing the whole GRAS domain, and the latter containing the PFYRE and SAW motifs of the GRAS domain in TaMOC1. The vectors were then transformed into yeast strain AH109 in liquid culture, which was diluted to an absorbance at 600 nm (A600) set at 1.0. Serial dilutions were inoculated onto tryptophan-, histidine- and adenine-negative synthetic dropout medium. The pGBKT7-vector was used as the negative control.

### Sample preparation and real-time quantitative PCR

In order to investigate the expression patterns of *TaMOC1,* tissue samples were collected from the roots (SR), tiller buds (TB) and leaf blades (SL) of seedlings, and roots (BR), internodes (BI), nodes (BN), leaf sheaths (BS), leaf blades (BL), and ears (BE) at late booting. Three kinds of tissue samples at various developmental stages were also collected, including tiller buds at the 4-leaf (4T), 5-leaf (5T), and 6-leaf (6T) and greening after winter (GT) stages, ears with lengths ranging from 2 to 10 cm (2E, 3E, 6E, 8E, and 10E), ears at the late booting (BE) and flowering (FE) stages, and grains at 4 (4G), 7 (7G), 14 (14G), and 21 (21G) days post-flowering.

Quantitative real-time PCR (qRT-PCR) was performed to determine the expression pattern of *TaMOC1*. Primer sequences amplifying wheat *Actin*, 5’-CTCCCTCACAACAACAACCGC-3’ and 5’-TACCAGGAACTTCCATACCAAC-3’, were used as internal controls to quantify relative transcript levels. Gene-specific qRT-PCR primers (5’-CACCGCTGTCACGAGCCT-3’ and 5’-TCACCCACTTCAGGAACGCT-3’) were designed from the *TaMOC1* cDNA sequence. qRT–PCR was performed in triplicate with an ABI Prism 7500 system using the SYBR Green PCR master mix kit (TaKaRa Biotechnology Co. Ltd, Dalian, China). To detect the transcription level of *TaMOC1* in different tissue samples, the expression of *TaMOC1* in the tissue sample with the lowest expression level was regarded as a standard, and corresponding formula was modified as 2^−ΔΔ*C*T^^55^. ΔΔ*C*_T_ = (*C*_T, Target_ − *C*_T, Actin_)_sample x_ − (*C*_T, Target_ − *C*_T, Actin_)_sample 0_. Sample 0 is the sample with the lowest expression level and sample x is any sample except sample 0. The qRT-PCR results were reported using a log scale (log2).

### SNP detection and functional marker development

Thirty-seven cultivars were initially chosen for detecting sequence differences in the *TaMOC1-A* coding and flanking regions sequences. Two haplotypes of *TaMOC1-A* were formed by three SNPs (G/A, G/A, G/T) and one indel (–/AG). A pair of primers (TaMAMF and TaMAMR), specific for the region flanking the indel was designed; the sequences were 5’-GTGGTAAGATATGATAGATGCAAGT-3’ and 5’-GAGAGGGAGGGAGAGTAGGTA-3’. PCR products were separated using polyacrylamide gel electrophoresis and silver staining to distinguish fragment lengths.

### Genetic mapping of *TaMOC1*

The functional marker TaMAMF/TaMAMR shows polymorphisms between two Chinese wheat cultivars Hanxuan 10 and Lumai 14, parents of the DH population, which possessed different haplotypes (*Hap*H and *Hap*L). The genetic linkage map of the DH population was established from the 150 DH lines using MAPMAKER/Exp version 3.0 software, consisted of 395 marker loci covering 3,904 cM with an average distance of 9.9 cM between adjacent markers^56^,^57^,^58^. The genotypes of the 150 DH lines derived from Hanxuan 10 and Lumai 14 were identified using the functional marker TaMAMF/TaMAMR. Based on the genotypic data of marker TaMAMF/TaMAMR and 395 marker loci, *TaMOC1* was mapped on the genetic linkage map of the DH population using the MAPMAKER/EXP version 3.0.

### Population structure and association analysis

Population structure was determined by STRUCTURE v2.3.2 using data from 209 whole-genome SSR markers^32^. A general linear model (GLM) was performed in TASSEL V2.1 for association analysis, which accounted for population structure (*Q*). Statistical analysis was conducted by SAS 8.01 software.

### Sequencing and evolutionary analyses

DNA polymorphism, nucleotide diversity (*π*) and linkage disequilibrium (LD) were analyzed by DnaSP 5.10 software. Population pairwise *F*_*st*_ values were calculated using Arlequin ver 3.1.

## Additional Information

**How to cite this article**: Zhang, B. *et al.* Novel function of a putative *MOC1* ortholog associated with spikelet number per spike in common wheat. *Sci. Rep.* **5**, 12211; doi: 10.1038/srep12211 (2015).

**Accession codes**: The GenBank accession number for *TaMOC1* is KR758748.

## Supplementary Material

Supplementary Information

## Figures and Tables

**Table 1:** *TaMOC1-7A* haplotype associations with agronomic traits in twelve environments.

Environment	**PH**		**SL**		**SN**		**GN**		**TGW**	
	***P*** **value**	***PVE*** **(%)**	***P*** **value**	***PVE*** **(%)**	***P*** **value**	***PVE*** **(%)**	***P*** **value**	***PVE*** **(%)**	***P*** **value**	***PVE*** **(%)**
E1	n.s.	—	n.s.	—	0.0087	4.37	n.s.	—	n.s.	—
E2	n.s.	—	n.s.	—	0.0065	3.29	n.s.	—	n.s.	—
E3	n.s.	—	n.s.	—	0.0015	4.04	n.s.	—	n.s.	—
E4	n.s.	—	n.s.	—	0.0043	3.93	0.0404	2.64	n.s.	—
E5	n.s.	—	n.s.	—	0.0155	4.21	0.0326	2.17	n.s.	—
E6	0.0209	3.41	n.s.	—	0.0183	2.57	n.s.	—	n.s.	—
E7	n.s.	—	n.s.	—	2.06E-04	6.00	n.s.	—	n.s.	—
E8	n.s.	—	n.s.	—	0.0201	2.61	n.s.	—	n.s.	—
E9	n.s.	—	n.s.	—	0.0012	4.66	n.s.	—	n.s.	—
E10	n.s.	—	n.s.	—	n.s.	—	n.s.	—	n.s.	—
E11	n.s.	—	n.s.	—	8.70E-04	4.64	0.0261	3.89	n.s.	—
E12	n.s.	—	n.s.	—	n.s.	—	n.s.	—	n.s.	—

PH: plant height; SL: spike length; SN: spikelet number per spike; GN: grain number per spike; TGW: 1000-grain weight; n.s.: not significant at *P* = 0.05; *PVE*: phenotypic variation explained. E1 to E12 indicate the environments at Changping in 2009 under drought stress (DS) and well-watered (WW) conditions, Changping in 2010 under DS and WW, Shunyi in 2010 under DS, WW, DS + heat stress (HS), and WW + HS, and Shunyi in 2011 under DS, WW, DS + HS and WW + HS, respectively.
