# Supplementary material for: Novel function of a putative MOC1 ortholog associated with spikelet number per spike in common wheat
Source: Sci Rep. 2015 Jul 22;5:12211. doi: 10.1038/srep12211 (PMC4510493; doi:10.1038/srep12211)
Supplement: Supplementary Information [file srep12211-s1.pdf]

Title

Novel function of a putative *MOC1* ortholog associated with spikelet number per spike in common wheat

Authors and addresses

First author: Bin Zhang

Corresponding author: Ruilian Jing

Order of Authors: Bin Zhang, Xia Liu, Weina Xu, Jianzhong Chang, Ang Li, Xinguo Mao, Xueyong Zhang, Ruilian Jing*

National Key Facility for Crop Gene Resources and Genetic Improvement / Institute of Crop Science, Chinese Academy of Agricultural Sciences, Beijing 100081, China.

Correspondence:

National Key Facility for Crop Gene Resources and Genetic Improvement / Institute of Crop Science, Chinese Academy of Agricultural Sciences, Beijing 100081, China

Tel/Fax: +86 (0)10 82105829

E-mail: jingruilian@caas.cn

Supplementary Information

Supplementary Fig. S1 Effects of *TaSnRK2.10* on 1000-grain weight (TGW) and spikelet number per spike (GN) in 12 environments. *TaSnRK2.10-C* and *TaSnRK2.10-G* are haplotypes (alleles) of *TaSnRK2.10.* See caption to Table 1 for description of environments. *, **, *** Significant at P = 0.05, 0.01 and 0.001, respectively. Bars indicate 2×SE. There was no significant difference in spikelet number per spike between *TaSnRK2.10-C* and *TaSnRK2.10-G*.

Supplementary Table S1 Combining favorable alleles of *TaMOC1-7A* and *TaSnRK2.10* contributing to spikelet number per spike (SN) and 1000-grain weight (TGW) in twelve environments

Supplementary Table S2 Thirty-seven cultivars with large variations in tiller number and spike-related traits.

Supplementary Table S3 Basic information of 262 accessions (names, origins and release dates) and sequence polymorphism assays of *TaMOC1-7A* and *TaSnRK2.10*

Supplementary Table S4 Basic information of 157 landrace accessions (names, origins and ecological zones) and sequence polymorphism assays of *TaMOC1-7A*

Supplementary Table S5 Basic information of 348 accessions (names, origins, ecological zones and release dates) and sequence polymorphism assays of *TaMOC1-7A*

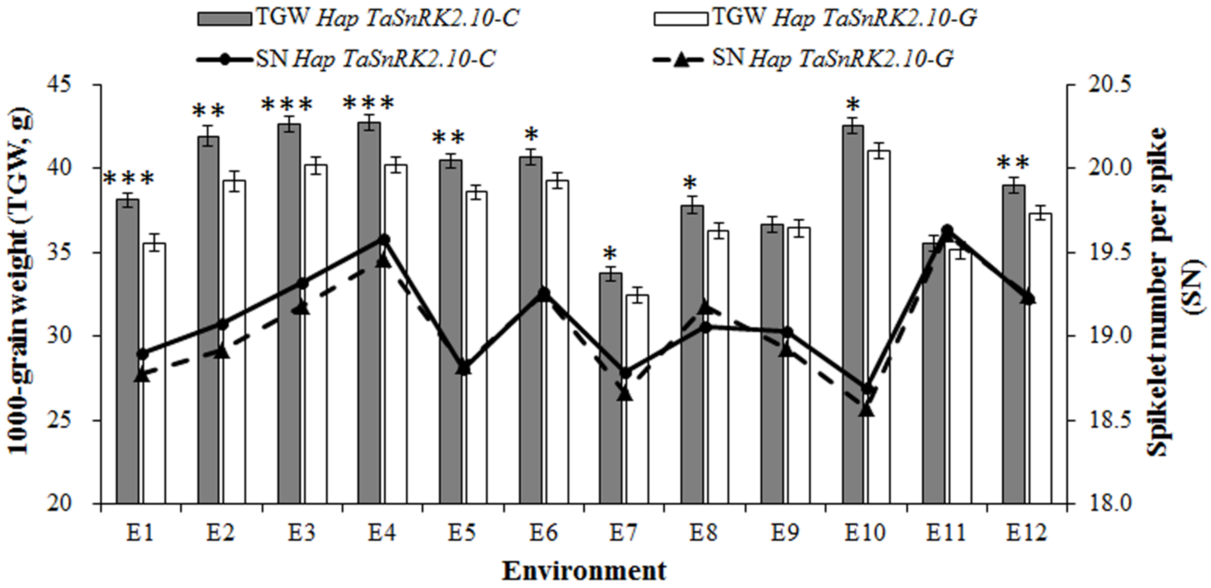

Supplementary Fig. S1 Effects of *TaSnRK2.10* on 1000-grain weight (TGW) and spikelet number per spike (GN) in 12 environments. *TaSnRK2.10-C* and *TaSnRK2.10-G* are haplotypes (alleles) of *TaSnRK2.10.* See caption to Table 1 for description of environments. *, **, *** Significant at P = 0.05, 0.01 and 0.001, respectively. Bars indicate 2×SE. There was no significant difference in spikelet number per spike between *TaSnRK2.10-C* and *TaSnRK2.10-G*.

Supplementary Table S1 Combining favorable alleles of *TaMOC1-7A* and *TaSnRK2.10* contributing to spikelet number per spike (SN) and 1000-grain weight (TGW) in twelve environments

Environment	No. of genes combined	SN [1] Mean ± SE (%)	Frequency (%)	TGW [1] Mean ± SE (%)	Frequency (%)
E1	2	19.2 ± 0.2 (a)	8.40	39.5 ± 0.9 (a)	8.78
	1	18.9 ± 0.1 (ab)	45.04	37.0 ± 0.5 (b)	45.04
	0	18.7 ± 0.1 (b)	40.84	35.9 ± 0.5 (b)	40.08
E2	2	19.2 ± 0.2 (a)	8.78	43.7 ± 0.9 (a)	8.40
	1	19.2 ± 0.1 (a)	45.42	40.9 ± 0.7 (b)	45.42
	0	18.7 ± 0.1 (a)	39.69	39.6 ± 0.7 (b)	39.31
E3	2	19.5 ± 0.2 (a)	8.78	44.4 ± 1.1 (a)	8.78
	1	19.4 ± 0.1 (ab)	46.18	41.4 ± 0.5 (b)	46.18
	0	19.0 ± 0.1 (b)	40.84	40.7 ± 0.6 (b)	41.22
E4	2	19.9 ± 0.2 (a)	8.78	44.0 ± 1.1 (a)	8.78
	1	19.6 ± 0.1 (ab)	45.42	41.5 ± 0.5 (b)	46.18
	0	19.3 ± 0.1 (b)	40.84	40.9 ± 0.5 (b)	41.22
E5	2	19.0 ± 0.2 (a)	8.78	41.8 ± 0.9 (a)	8.78
	1	18.9 ± 0.1 (a)	46.18	39.6 ± 0.4 (b)	46.18
	0	18.7 ± 0.1 (a)	41.22	38. 9 ± 0.5 (b)	41.22
E6	2	19.5 ± 0.2 (a)	8.78	41.9 ± 1.0 (a)	8.78
	1	19.3 ± 0.1 (a)	40.46	40.2 ± 0.5 (ab)	46.18
	0	19.1 ± 0.1 (a)	38.55	39.3 ± 0.5 (b)	41.22
E7	2	19.1 ± 0.2 (a)	8.78	35.3 ± 1.0 (a)	8.78
	1	18.9 ± 0.1 (ab)	45.80	33.0 ± 0.4 (b)	46.18
	0	18.5 ± 0.1 (b)	41.22	32.7 ± 0.5 (b)	41.22
E8	2	19.2 ± 0.3 (a)	8.78	39.6 ± 0.9 (a)	8.78
	1	19.3 ± 0.2 (a)	43.51	37.1 ± 0.5 (b)	46.18
	0	19.0 ± 0.2 (a)	40.46	36.4 ± 0.6 (b)	41.22

See caption to Table 1 for description of environments. [1]Values with different letters in the same group are significantly different at $P = 0.05$.

Supplementary Table S2. Thirty-seven cultivars with large variations in tiller number and spike-related traits

No.	Name	Origin
1	TAM-110	USA
2	An85 zhong124-1	China (Beijing)
3	Dan R8108	China (Beijing)
4	Yuandong 847	China (Beijing)
5	Beijing 10	China (Beijing)
6	Jinghe 8922	China (Beijing)
7	Zhongyou 9507	China (Beijing)
8	Cangmai 6005	China (Hebei)
9	Hengmai 2	China (Hebei)
10	Handan 6050	China (Hebei)
11	Heng 7228	China (Hebei)
12	Hengguan 35	China (Hebei)
13	Shi 4185	China (Hebei)
14	Shimai 12	China (Hebei)
15	Bainong 3217	China (Henan)
16	Yanzhanyihao	China (Henan)
17	Yanzhan 4110	China (Henan)
18	Yumai 29	China (Henan)
19	Yumai 38	China (Henan)
20	Yumai 48	China (Henan)
21	Huaimai 18	China (Jiangsu)
22	Shuangfengshou	China (Shaanxi)
23	Lumai 14	China (Shandong)
24	Shandongyoumai 2	China (Shandong)
25	Chang 6359	China (Shanxi)
26	Jinmai 16	China (Shanxi)
27	Jinmai 25	China (Shanxi)
28	Jinmai 39	China (Shanxi)
29	Hanxuan 10	China (Shanxi)
30	Jinmai 51	China (Shanxi)
31	Linfeng 615	China (Shanxi)
32	Taiyuan 633	China (Shanxi)
33	Yunhan 20410	China (Shanxi)
34	PANDAS	Italy
35	Opata 85	CIMMYT
36	W7984	CIMMYT
37	Neixiang 188	China

Supplementary Table S3. Basic information of 262 accessions (names, origins and released dates) and their sequence polymorphism assays of *TaMOC1-7A* and *TaSnRK2.10*

No.	Name	Origin	Decade	*TaMOC1-7A* Haplotype	*TaSnRK2.10* SNP
1	Bima 1	China (Shaanxi)	Pre-1960	*Hap* L	*TaSnRK2.10-G*
2	Xinong 6028	China (Shaanxi)	Pre-1960	*Hap* L	*TaSnRK2.10-C*
3	Huabei 187	China (Beijing)	Pre-1960	*Hap* L	*TaSnRK2.10-C*
4	Nongda 183	China (Beijing)	Pre-1960	*HaP* H	*TaSnRK2.10-G*
5	Nongda 20074	China (Beijing)	Pre-1960	*Hap* L	*TaSnRK2.10-C*
6	Nongda 36	China (Beijing)	Pre-1960	*HaP* H	*TaSnRK2.10-C*
7	Yanda 1817	China (Beijing)	Pre-1960	*HaP* H	*TaSnRK2.10-G*
8	Shijiazhuang 407	China (Hebei)	Pre-1960	*Hap* L	*TaSnRK2.10-G*
9	Shite 14	China (Hebei)	Pre-1960	*Hap* L	*TaSnRK2.10-C*
10	Early Premium	USA	Pre-1960	*Hap* L	*TaSnRK2.10-C*
11	Triumph	USA	Pre-1960	*Hap* L	*TaSnRK2.10-C*
12	Beijing 10	China (Beijing)	1960S	*Hap* L	*TaSnRK2.10-C*
13	Jingyan85 jian28	China (Beijing)	1960S	*Hap* L	*TaSnRK2.10-C*
14	Keyi 29	China (Beijing)	1960s	*Hap* L	*TaSnRK2.10-G*
15	Nongda 155	China (Beijing)	1960S	*Hap* L	*TaSnRK2.10-C*
16	Nongda 311	China (Beijing)	1960S	*Hap* L	*TaSnRK2.10-C*
17	Nongda 33	China (Beijing)	1960S	*Hap* L	*TaSnRK2.10-G*
18	Zhongsu 68	China (Beijing)	1960S	*HaP* H	*TaSnRK2.10-G*
19	Xifeng 9	China (Gansu)	1960S	*Hap* L	*TaSnRK2.10-G*
20	Zhengzhou 24	China (Henan)	1960S	*Hap* L	*TaSnRK2.10-C*
21	Aiganzao	China (Jiangsu)	1960S	*Hap* L	*TaSnRK2.10-G*
22	Xuzhou 6	China (Jiangsu)	1960S	*Hap* L	*TaSnRK2.10-C*
23	Fengchan 1	China (Shaanxi)	1960S	*Hap* L	*TaSnRK2.10-C*

24	Fengchan 3	China (Shaanxi)	1960S	*Hap* L	*TaSnRK2.10-G*
25	Fuzhuang 30	China (Shaanxi)	1960S	*Hap* L	*TaSnRK2.10-G*
26	Jinguang	China (Shaanxi)	1960S	*Hap* L	*TaSnRK2.10-G*
27	Qingchun 1	China (Shaanxi)	1960S	*Hap* L	*TaSnRK2.10-G*
28	Qingchun 2	China (Shaanxi)	1960S	*Hap* L	*TaSnRK2.10-G*
29	Shaannong 1	China (Shaanxi)	1960S	*Hap* L	*TaSnRK2.10-G*
30	Shaannong 2	China (Shaanxi)	1960S	*Hap* L	*TaSnRK2.10-G*
31	Jinan 10	China (Shandong)	1960S	*Hap* L	*TaSnRK2.10-G*
32	Jinan 2	China (Shandong)	1960S	*Hap* L	*TaSnRK2.10-G*
33	Jining 3	China (Shandong)	1960S	*Hap* L	*TaSnRK2.10-G*
34	Changzhi 620	China (Shanxi)	1960S	*Hap* L	*TaSnRK2.10-G*
35	Hanxuan 1	China (Shanxi)	1960S	*Hap* L	*TaSnRK2.10-C*
36	Hanxuan 2	China (Shanxi)	1960S	*Hap* L	*TaSnRK2.10-G*
37	Jinmai 13	China (Shanxi)	1960S	*HaP* H	*TaSnRK2.10-G*
38	Hanxuan 10	China (Shanxi)	1960S	*HaP* H	*TaSnRK2.10-G*
39	An85 zhong124-1	China (Beijing)	1970S	*Hap* L	*TaSnRK2.10-C*
40	An86 zhong17	China (Beijing)	1970S	*Hap* L	*TaSnRK2.10-C*
41	Dan R8043	China (Beijing)	1970S	*Hap* L	*TaSnRK2.10-C*
42	Dan R8093	China (Beijing)	1970S	*Hap* L	*TaSnRK2.10-G*
43	Dan R8108	China (Beijing)	1970S	*Hap* L	*TaSnRK2.10-G*
44	Dan R8194	China (Beijing)	1970S	*Hap* L	*TaSnRK2.10-C*
45	Dan R9062	China (Beijing)	1970S	*Hap* L	*TaSnRK2.10-G*
46	Hongliang 4	China (Beijing)	1970S	*Hap* L	*TaSnRK2.10-G*
47	Jian 26	China (Beijing)	1970S	*Hap* L	*TaSnRK2.10-C*
48	Jingdong82 dong307	China (Beijing)	1970S	*Hap* L	*TaSnRK2.10-G*
49	Jingdong83 dong65	China (Beijing)	1970S	*Hap* L	*TaSnRK2.10-C*

50	Jingnong 79-15	China (Beijing)	1970S	*Hap* L	*TaSnRK2.10-C*
51	Jingnong 84-6789	China (Beijing)	1970S	*HaP* H	*TaSnRK2.10-C*
52	Jingnong80 jian107	China (Beijing)	1970S	*Hap* L	*TaSnRK2.10-C*
53	Jingpin 11	China (Beijing)	1970S	*Hap* L	*TaSnRK2.10-G*
54	Jingpin 30	China (Beijing)	1970S	*Hap* L	*TaSnRK2.10-G*
55	Jingshuang 2	China (Beijing)	1970S	*Hap* L	*TaSnRK2.10-G*
56	Jingxuan 20	China (Beijing)	1970S	*Hap* L	*TaSnRK2.10-C*
57	Jingxuan 25	China (Beijing)	1970S	*Hap* L	*TaSnRK2.10-C*
58	Keyi 26	China (Beijing)	1970S	*HaP* H	*TaSnRK2.10-G*
59	Nongda 81146	China (Beijing)	1970S	*Hap* L	*TaSnRK2.10-C*
60	Xiaoshan 8	China (Beijing)	1970S	*Hap* L	*TaSnRK2.10-C*
61	Yuandong 834	China (Beijing)	1970S	*Hap* L	*TaSnRK2.10-C*
62	Yuandong 847	China (Beijing)	1970S	*Hap* L	*TaSnRK2.10-G*
63	Yuandong 856	China (Beijing)	1970S	*Hap* L	*TaSnRK2.10-C*
64	Zaosui 21	China (Beijing)	1970S	*Hap* L	*TaSnRK2.10-C*
65	Zaosui 65	China (Beijing)	1970S	*Hap* L	*TaSnRK2.10-C*
66	Zaosui 66	China (Beijing)	1970S	*Hap* L	*TaSnRK2.10-C*
67	Zhong 7902	China (Beijing)	1970S	*Hap* L	*TaSnRK2.10-C*
68	Zhong 8502	China (Beijing)	1970S	*Hap* L	*TaSnRK2.10-C*
69	Zhongda 86 - jian 2	China (Beijing)	1970S	*Hap* L	*TaSnRK2.10-C*
70	Zhongzuo 60064	China (Beijing)	1970S	*Hap* L	*TaSnRK2.10-C*
71	Zhongzuo 60115	China (Beijing)	1970S	*Hap* L	*TaSnRK2.10-C*
72	Qingfeng 1	China (Gansu)	1970S	*Hap* L	*TaSnRK2.10-G*
73	Zhangdong 29	China (Gansu)	1970S	*Hap* L	*TaSnRK2.10-C*
74	Hengmai 2	China (Hebei)	1970S	*Hap* L	*TaSnRK2.10-G*
75	Jimai 6	China (Hebei)	1970S	*Hap* L	*TaSnRK2.10-G*

76	Jimai 9	China (Hebei)	1970S	*Hap* L	*TaSnRK2.10-G*
77	Huaishu 10	China (Jiangsu)	1970S	*Hap* L	*TaSnRK2.10-C*
78	Dali 1	China (Shaanxi)	1970S	*Hap* L	*TaSnRK2.10-G*
79	Dali 52	China (Shaanxi)	1970S	*Hap* L	*TaSnRK2.10-G*
80	Luonong 10	China (Shaanxi)	1970S	*Hap* L	*TaSnRK2.10-C*
81	Shaanhe 6	China (Shaanxi)	1970S	*Hap* L	*TaSnRK2.10-C*
82	Shuangfengshou	China (Shaanxi)	1970S	*Hap* L	*TaSnRK2.10-G*
83	Yanan 15	China (Shaanxi)	1970S	*Hap* L	*TaSnRK2.10-C*
84	Changle 5	China (Shandong)	1970S	Unknown	*TaSnRK2.10-G*
85	Jinan 13	China (Shandong)	1970S	*Hap* L	*TaSnRK2.10-C*
86	Changzhi 516	China (Shanxi)	1970S	*HaP* H	*TaSnRK2.10-G*
87	Hanxuan 11	China (Shanxi)	1970S	*Hap* L	*TaSnRK2.10-G*
88	Hanxuan 12	China (Shanxi)	1970S	*Hap* L	*TaSnRK2.10-G*
89	Jinmai 17	China (Shanxi)	1970S	*Hap* L	*TaSnRK2.10-G*
90	Jinmai 25	China (Shanxi)	1970S	*Hap* L	*TaSnRK2.10-G*
91	Mingxian 169	China (Shanxi)	1970s	*HaP* H	*TaSnRK2.10-G*
92	Pingyang 348	China (Shanxi)	1970S	*Hap* L	*TaSnRK2.10-G*
93	Lovrin 10	Romania	1970S	*Hap* L	*TaSnRK2.10-C*
94	Beijing 14	China (Beijing)	1980S	*Hap* L	*TaSnRK2.10-G*
95	Beijing 837	China (Beijing)	1980S	*Hap* L	*TaSnRK2.10-C*
96	Beinong 2	China (Beijing)	1980S	*Hap* L	*TaSnRK2.10-C*
97	Dongxie 2	China (Beijing)	1980S	*Hap* L	*TaSnRK2.10-G*
98	Fengkang 13	China (Beijing)	1980S	*Hap* L	*TaSnRK2.10-G*
99	Jinghe 8922	China (Beijing)	1980S	*Hap* L	*TaSnRK2.10-G*
100	Jinghua 1	China (Beijing)	1980S	*Hap* L	*TaSnRK2.10-C*
101	Jingpin 3	China (Beijing)	1980S	*Hap* L	*TaSnRK2.10-C*

102	Jingshuang 16	China (Beijing)	1980S	*Hap* L	*TaSnRK2.10-C*
103	Yuandong 3	China (Beijing)	1980S	*Hap* L	*TaSnRK2.10-C*
104	Jin 2148 - 7	China (Fujian)	1980S	*HaP* H	*TaSnRK2.10-G*
105	Pingliang 35	China (Gansu)	1980S	*Hap* L	*TaSnRK2.10-C*
106	Xifeng 16	China (Gansu)	1980S	*HaP* H	*TaSnRK2.10-G*
107	Hengshui 6404	China (Hebei)	1980S	*Hap* L	*TaSnRK2.10-G*
108	Ji 92 - 5203	China (Hebei)	1980s	*HaP* H	*TaSnRK2.10-G*
109	Jimai 10	China (Hebei)	1980S	*Hap* L	*TaSnRK2.10-C*
110	Jimai 18	China (Hebei)	1980S	*Hap* L	*TaSnRK2.10-G*
111	Jimai 22	China (Hebei)	1980S	*HaP* H	*TaSnRK2.10-C*
112	Jimai 29	China (Hebei)	1980S	*Hap* L	*TaSnRK2.10-G*
113	Jimaiyihao	China (Hebei)	1980S	*Hap* L	*TaSnRK2.10-G*
114	Bainong 3217	China (Henan)	1980S	*Hap* L	*TaSnRK2.10-G*
115	Yumai 13	China (Henan)	1980S	*Hap* L	*TaSnRK2.10-G*
116	Yumai 2	China (Henan)	1980S	*Hap* L	*TaSnRK2.10-G*
117	Yumai 8	China (Henan)	1980S	*Hap* L	*TaSnRK2.10-G*
118	Xuzhou 21	China (Jiangsu)	1980S	*Hap* L	*TaSnRK2.10-G*
119	Ningchun 4	China (Ningxia)	1980S	*Hap* L	*TaSnRK2.10-C*
120	Baolin 9	China (Shaanxi)	1980S	*Hap* L	*TaSnRK2.10-G*
121	Changwu 131	China (Shaanxi)	1980S	*HaP* H	*TaSnRK2.10-C*
122	Qinmai 3	China (Shaanxi)	1980S	*Hap* L	*TaSnRK2.10-C*
123	Qinmai 7	China (Shaanxi)	1980S	*Hap* L	*TaSnRK2.10-C*
124	Shaannong 7859	China (Shaanxi)	1980S	*Hap* L	*TaSnRK2.10-C*
125	Weimai 4	China (Shaanxi)	1980S	*Hap* L	*TaSnRK2.10-G*
126	Xi'an 8	China (Shaanxi)	1980S	Unknown	*TaSnRK2.10-C*
127	AimengniuⅣxing (8057)	China (Shandong)	1980S	Unknown	Unknown

128	Lu 215953	China (Shandong)	1980S	*Hap* L	*TaSnRK2.10-C*
129	Lumai 1	China (Shandong)	1980S	*Hap* L	*TaSnRK2.10-C*
130	Lumai 3	China (Shandong)	1980S	*HaP* H	*TaSnRK2.10-G*
131	Lumai 5	China (Shandong)	1980S	*HaP* H	*TaSnRK2.10-C*
132	Lumai 8	China (Shandong)	1980S	*Hap* L	*TaSnRK2.10-G*
133	Taiyuan 633	China (Shanxi)	1980S	*HaP* H	*TaSnRK2.10-C*
134	Wanmai 19	China (Anhui)	1990S	*Hap* L	*TaSnRK2.10-C*
135	Beijing 8686	China (Beijing)	1990S	*Hap* L	*TaSnRK2.10-C*
136	Beijing 8694	China (Beijing)	1990S	*Hap* L	*TaSnRK2.10-G*
137	Fengyou 5	China (Beijing)	1990S	*Hap* L	*TaSnRK2.10-G*
138	Jing 411	China (Beijing)	1990S	Unknown	*TaSnRK2.10-C*
139	Jingdong 8	China (Beijing)	1990S	*Hap* L	*TaSnRK2.10-C*
140	Lunkang 7	China (Beijing)	1990S	*Hap* L	*TaSnRK2.10-C*
141	Nongda 146	China (Beijing)	1990S	*Hap* L	*TaSnRK2.10-C*
142	Xiaoyan 54	China (Beijing)	1990S	*Hap* L	*TaSnRK2.10-C*
143	Zhongda 91 - pin 9	China (Beijing)	1990S	*Hap* L	*TaSnRK2.10-C*
144	Zhongda 92 - jian 49	China (Beijing)	1990S	*Hap* L	*TaSnRK2.10-C*
145	Zhongda 92 - pin 8	China (Beijing)	1990S	*Hap* L	*TaSnRK2.10-G*
146	Zhongmai 9	China (Beijing)	1990S	*Hap* L	*TaSnRK2.10-C*
147	Longjian 196	China (Gansu)	1990S	*Hap* L	*TaSnRK2.10-G*
148	Qingshan 843	China (Gansu)	1990S	*Hap* L	*TaSnRK2.10-C*
149	Xifeng 20	China (Gansu)	1990S	*Hap* L	*TaSnRK2.10-G*
150	Cangmai 6001	China (Hebei)	1990S	*Hap* L	*TaSnRK2.10-C*
151	Han 4589	China (Hebei)	1990s	*Hap* L	*TaSnRK2.10-C*
152	Jimai 26	China (Hebei)	1990S	*HaP* H	*TaSnRK2.10-C*
153	Jimai 30	China (Hebei)	1990S	*HaP* H	*TaSnRK2.10-G*

154	Jimai 32	China (Hebei)	1990S	*Hap* L	*TaSnRK2.10-C*
155	Jimai 41	China (Hebei)	1990S	*Hap* L	*TaSnRK2.10-G*
156	Shi 4185	China (Hebei)	1990S	*HaP* H	*TaSnRK2.10-G*
157	Luoyang 8628	China (Henan)	1990S	*Hap* L	*TaSnRK2.10-G*
158	Yanzhanyihao	China (Henan)	1990S	*Hap* L	*TaSnRK2.10-G*
159	Yumai 18	China (Henan)	1990S	*Hap* L	*TaSnRK2.10-C*
160	Yumai 38	China (Henan)	1990S	*HaP* H	*TaSnRK2.10-G*
161	Yumai 47	China (Henan)	1990S	*HaP* H	*TaSnRK2.10-G*
162	Yumai 48	China (Henan)	1990S	*Hap* L	*TaSnRK2.10-G*
163	Huaimai 18	China (Jiangsu)	1990S	*Hap* L	*TaSnRK2.10-G*
164	Baomai 5	China (Shaanxi)	1990S	*Hap* L	*TaSnRK2.10-G*
165	Changwu 89 (1) 3 - 4	China (Shaanxi)	1990S	*HaP* H	*TaSnRK2.10-C*
166	Shaan 229	China (Shaanxi)	1990S	*Hap* L	*TaSnRK2.10-C*
167	Shaanhan 8675	China (Shaanxi)	1990S	*Hap* L	*TaSnRK2.10-G*
168	Shaanyou 225	China (Shaanxi)	1990S	*HaP* H	*TaSnRK2.10-C*
169	Shaanyou 225-9	China (Shaanxi)	1990S	*Hap* L	*TaSnRK2.10-C*
170	Xinong 1043	China (Shaanxi)	1990S	*HaP* H	*TaSnRK2.10-C*
171	Lude 1	China (Shandong)	1990S	*Hap* L	*TaSnRK2.10-C*
172	Lumai 14	China (Shandong)	1990S	*Hap* L	*TaSnRK2.10-G*
173	Lumai 15	China (Shandong)	1990S	*HaP* H	*TaSnRK2.10-G*
174	Lumai 17	China (Shandong)	1990S	*Hap* L	*TaSnRK2.10-C*
175	Lumai19	China (Shandong)	1990S	*HaP* H	*TaSnRK2.10-C*
176	Shannongfu 63	China (Shandong)	1990S	*Hap* L	*TaSnRK2.10-G*
177	Yan 881414	China (Shandong)	1990S	*Hap* L	*TaSnRK2.10-G*
178	Xiaoyan 22	China (Shannxi)	1990S	*Hap* L	*TaSnRK2.10-G*
179	Jinmai 16	China (Shanxi)	1990S	*HaP* H	*TaSnRK2.10-G*

180	Jinmai 39	China (Shanxi)	1990S	*HaP* H	*TaSnRK2.10-G*
181	Jinmai 44	China (Shanxi)	1990S	*Hap* L	*TaSnRK2.10-G*
182	Jinmai 47	China (Shanxi)	1990S	*Hap* L	*TaSnRK2.10-G*
183	Jinmai 50	China (Shanxi)	1990S	*Hap* L	*TaSnRK2.10-G*
184	Jinmai 51	China (Shanxi)	1990S	*HaP* H	*TaSnRK2.10-G*
185	Jinmai 53	China (Shanxi)	1990S	*HaP* H	*TaSnRK2.10-C*
186	Jinmai 54	China (Shanxi)	1990S	*Hap* L	*TaSnRK2.10-G*
187	Jinmai 57	China (Shanxi)	1990S	*Hap* L	*TaSnRK2.10-C*
188	Linhan 6105	China (Shanxi)	1990S	*Hap* L	*TaSnRK2.10-G*
189	Linhan 917	China (Shanxi)	1990S	*Hap* L	*TaSnRK2.10-G*
190	Linhan 935	China (Shanxi)	1990S	*Hap* L	*TaSnRK2.10-G*
191	PANDAS	Italy	1990S	*Hap* L	*TaSnRK2.10-G*
192	SALGEMMA	Italy	1990S	*Hap* L	*TaSnRK2.10-C*
193	Drysdale	Australia	2000S	*Hap* L	*TaSnRK2.10-C*
194	Rees	Australia	2000S	*Hap* L	*TaSnRK2.10-C*
195	H 89	China (Beijing)	2000S	Unknown	Unknown
196	Lunxuan 987	China (Beijing)	2000S	*Hap* L	*TaSnRK2.10-G*
197	Nongda 135	China (Beijing)	2000S	*HaP* H	*TaSnRK2.10-C*
198	Xiaoyan 81	China (Beijing)	2000S	*HaP* H	*TaSnRK2.10-G*
199	Zhonghan 110	China (Beijing)	2000S	*Hap* L	*TaSnRK2.10-G*
200	Zhongyou 9507	China (Beijing)	2000S	*Hap* L	Unknown
201	Longjian 294	China (Gansu)	2000S	*Hap* L	*TaSnRK2.10-G*
202	Heng 5229	China (Hebei)	2000S	*HaP* H	*TaSnRK2.10-C*
203	Heng 7228	China (Hebei)	2000S	Unknown	*TaSnRK2.10-C*
204	Heng95 guan26	China (Hebei)	2000S	*HaP* H	*TaSnRK2.10-C*
205	Hengguan 35	China (Hebei)	2000S	*HaP* H	*TaSnRK2.10-C*

206	Hengyou 18	China (Hebei)	2000S	*HaP* H	*TaSnRK2.10-C*
207	Shijiazhuang 8	China (Hebei)	2000S	*HaP* H	*TaSnRK2.10-C*
208	Shimai 12	China (Hebei)	2000S	*HaP* H	*TaSnRK2.10-C*
209	Shimai 13	China (Hebei)	2000S	*HaP* H	*TaSnRK2.10-C*
210	Luohan 2	China (Henan)	2000S	*Hap* L	*TaSnRK2.10-G*
211	Luohan 3	China (Henan)	2000S	*Hap* L	*TaSnRK2.10-G*
212	Luohan 6	China (Henan)	2000S	*Hap* L	*TaSnRK2.10-G*
213	Yumai 49	China (Henan)	2000S	*Hap* L	*TaSnRK2.10-G*
214	Zhoumai 18	China (Henan)	2000S	*Hap* L	*TaSnRK2.10-C*
215	Xinong 928	China (Shaanxi)	2000S	*HaP* H	*TaSnRK2.10-C*
216	Xinong 979	China (Shaanxi)	2000S	*Hap* L	*TaSnRK2.10-C*
217	Jimai 21	China (Shandong)	2000S	*Hap* L	*TaSnRK2.10-G*
218	Lumai 21	China (Shandong)	2000S	*Hap* L	*TaSnRK2.10-G*
219	Lumai 23	China (Shandong)	2000S	*Hap* L	*TaSnRK2.10-C*
220	Taishan 23	China (Shandong)	2000S	*Hap* L	*TaSnRK2.10-G*
221	Taishan 24	China (Shandong)	2000S	*Hap* L	*TaSnRK2.10-G*
222	Yannong 21	China (Shandong)	2000S	*Hap* L	*TaSnRK2.10-G*
223	Chang 4640	China (Shanxi)	2000S	*Hap* L	*TaSnRK2.10-C*
224	Chang 4738	China (Shanxi)	2000S	*Hap* L	*TaSnRK2.10-C*
225	Chang 6154	China (Shanxi)	2000S	*Hap* L	*TaSnRK2.10-C*
226	Chang 6359	China (Shanxi)	2000S	*Hap* L	*TaSnRK2.10-C*
227	Chang 6452	China (Shanxi)	2000S	*HaP* H	*TaSnRK2.10-C*
228	Chang 6878	China (Shanxi)	2000S	*Hap* L	*TaSnRK2.10-G*
229	Jinmai 63	China (Shanxi)	2000S	*Hap* L	*TaSnRK2.10-C*
230	Jinmai 68	China (Shanxi)	2000S	*Hap* L	*TaSnRK2.10-G*
231	Jinmai 72	China (Shanxi)	2000S	*HaP* H	*TaSnRK2.10-G*

232	Jinmai 79	China (Shanxi)	2000S	*Hap* L	*TaSnRK2.10-G*
233	Jinnong 207	China (Shanxi)	2000S	*Hap* L	*TaSnRK2.10-C*
234	Jintai 170	China (Shanxi)	2000S	*Hap* L	*TaSnRK2.10-C*
235	Linfeng 3	China (Shanxi)	2000S	Unknown	*TaSnRK2.10-G*
236	Linfeng 615	China (Shanxi)	2000S	*Hap* L	*TaSnRK2.10-C*
237	Linhan 6	China (Shanxi)	2000S	*Hap* L	*TaSnRK2.10-G*
238	Linkang 5108	China (Shanxi)	2000S	*HaP* H	*TaSnRK2.10-G*
239	Yunhan 20410	China (Shanxi)	2000S	*Hap* L	*TaSnRK2.10-G*
240	Yunhan 21 - 30	China (Shanxi)	2000S	*Hap* L	*TaSnRK2.10-G*
241	Yunhan 22 - 33	China (Shanxi)	2000S	*Hap* L	*TaSnRK2.10-C*
242	Yunhan 23 - 35	China (Shanxi)	2000S	*Hap* L	*TaSnRK2.10-G*
243	Baiqimai	China (Gansu)	Landrace	*Hap* L	*TaSnRK2.10-G*
244	Bawangbian	China (Hebei)	Landrace	*Hap* L	*TaSnRK2.10-G*
245	Heimangmai	China (Hebei)	Landrace	*Hap* L	*TaSnRK2.10-C*
246	Hulutou	China (Hebei)	Landrace	*HaP* H	*TaSnRK2.10-G*
247	Silenghonghulutou	China (Hebei)	Landrace	*Hap* L	*TaSnRK2.10-G*
248	Baicaomai	China (Henan)	Landrace	*Hap* L	*TaSnRK2.10-G*
249	Mazhamai	China (Shaanxi)	Landrace	*Hap* L	*TaSnRK2.10-G*
250	Baitutou	China (Shandong)	Landrace	*Hap* L	*TaSnRK2.10-G*
251	Hongheshang	China (Shanxi)	Landrace	*Hap* L	*TaSnRK2.10-G*
252	Xiaobaimai	China (Shanxi)	Landrace	*HaP* H	*TaSnRK2.10-G*
253	Yuansheng 215	China (Beijing)	Unknown	Unknown	Unknown
254	Canzhouxiaomai	China (Hebei)	Unknown	*HaP* H	*TaSnRK2.10-C*
255	Gaoyou 504	China (Hebei)	Unknown	Unknown	Unknown
256	Handan 6050	China (Hebei)	Unknown	Unknown	Unknown
257	Luomai 9769	China (Henan)	Unknown	Unknown	Unknown

258	Luoyang 9048	China (Henan)	Unknown	Unknown	Unknown
259	Shaanzi 1869	China (Shaanxi)	Unknown	Unknown	Unknown
260	Linfeng 518	China (Shanxi)	Unknown	Unknown	Unknown
261	Yunhan 2028	China (Shanxi)	Unknown	*Hap* L	*TaSnRK2.10-C*
262	TAM-110	USA	Unknown	Unknown	Unknown

Supplementary Table S4. Basic information of 157 landrace accessions (names, origins and ecological zones) and their sequence polymorphism assays of *TaMOC1-7A*

No.	Name	Origin	Ecological Zones	*TaMOC1-7A* Haplotype
1	Chanbuzhi	China (Anhui)	Middle and Lower Yangtze Valleys Autumn-Sown Spring Wheat Zone	*Hap* H
2	Dahuangpi	China (Anhui)	Middle and Lower Yangtze Valleys Autumn-Sown Spring Wheat Zone	*Hap* L
3	Jiangxizao	China (Henan)	Middle and Lower Yangtze Valleys Autumn-Sown Spring Wheat Zone	*Hap* H
4	Xianmai	China (Henan)	Middle and Lower Yangtze Valleys Autumn-Sown Spring Wheat Zone	*Hap* L
5	Chongyanghongmai 1	China (Hubei)	Middle and Lower Yangtze Valleys Autumn-Sown Spring Wheat Zone	*Hap* L
6	Honghuazao	China (Hubei)	Middle and Lower Yangtze Valleys Autumn-Sown Spring Wheat Zone	*Hap* L
7	Sankecun	China (Hubei)	Middle and Lower Yangtze Valleys Autumn-Sown Spring Wheat Zone	*Hap* L
8	Heshangmai	China (Hunan)	Middle and Lower Yangtze Valleys Autumn-Sown Spring Wheat Zone	*Hap* H
9	Nuomai	China (Hunan)	Middle and Lower Yangtze Valleys Autumn-Sown Spring Wheat Zone	*Hap* L
10	Chejianzi	China (Jiangsu)	Middle and Lower Yangtze Valleys Autumn-Sown Spring Wheat Zone	*Hap* H

11	Jiangdongmen	China (Jiangsu)	Middle and Lower Yangtze Valleys Autumn-Sown Spring Wheat Zone	*Hap* H
12	Liuzhutou	China (Jiangsu)	Middle and Lower Yangtze Valleys Autumn-Sown Spring Wheat Zone	*Hap* H
13	Mangxiaomai	China (Jiangsu)	Middle and Lower Yangtze Valleys Autumn-Sown Spring Wheat Zone	*Hap* H
14	Wangshuibai	China (Jiangsu)	Middle and Lower Yangtze Valleys Autumn-Sown Spring Wheat Zone	*Hap* L
15	Zaowutian	China (Jiangsu)	Middle and Lower Yangtze Valleys Autumn-Sown Spring Wheat Zone	*Hap* H
16	Paozimai	China (Jiangxi)	Middle and Lower Yangtze Valleys Autumn-Sown Spring Wheat Zone	*Hap* L
17	Shuilizhan	China (Jiangxi)	Middle and Lower Yangtze Valleys Autumn-Sown Spring Wheat Zone	*Hap* L
18	Wuyuanmai	China (Jiangxi)	Middle and Lower Yangtze Valleys Autumn-Sown Spring Wheat Zone	*Hap* H
19	Baipu	China (Zhejiang)	Middle and Lower Yangtze Valleys Autumn-Sown Spring Wheat Zone	*Hap* L
20	Huangshuibai	China (Zhejiang)	Middle and Lower Yangtze Valleys Autumn-Sown Spring Wheat Zone	*Hap* H
21	Lanxizaoxiaomai	China (Zhejiang)	Middle and Lower Yangtze Valleys Autumn-Sown Spring Wheat Zone	*Hap* H
22	Zaoxiaomai	China (Zhejiang)	Middle and Lower Yangtze Valleys Autumn-Sown Spring Wheat Zone	*Hap* H
23	Zhumaoyuanzitou	China (Zhejiang)	Middle and Lower Yangtze Valleys Autumn-Sown Spring Wheat Zone	*Hap* H

24	Baiyoumai	China (Heilongjiang)	Northeastern Spring Wheat Zone	*Hap* L
25	Daqingmang	China (Heilongjiang)	Northeastern Spring Wheat Zone	*Hap* L
26	Guangtou	China (Heilongjiang)	Northeastern Spring Wheat Zone	*Hap* L
27	Yangmai	China (Heilongjiang)	Northeastern Spring Wheat Zone	*Hap* L
28	Huoqiu	China (Liaoning)	Northeastern Spring Wheat Zone	*Hap* H
29	Donghuachunmai	Unknown	Northeastern Spring Wheat Zone	*Hap* H
30	Dabaipi	China (Hebei)	Northern Spring Wheat Zone	*Hap* L
31	Hongpixiaomai	China (Neimenggu)	Northern Spring Wheat Zone	*Hap* L
32	Huoliaomai	China (Neimenggu)	Northern Spring Wheat Zone	*Hap* L
33	Xiaobaimai	China (Neimenggu)	Northern Spring Wheat Zone	*Hap* L
34	Xiaohongpi	China (Neimenggu)	Northern Spring Wheat Zone	*Hap* L
35	Chunxiaomai	China (Shannxi)	Northern Spring Wheat Zone	*Hap* H
36	Honglidangnianlao	China (Shannxi)	Northern Spring Wheat Zone	*Hap* L
37	Dahongmai	China (Shanxi)	Northern Spring Wheat Zone	*Hap* L
38	Dingxingzhai	China (Shanxi)	Northern Spring Wheat Zone	*Hap* L
39	Baiqimai	China (Gansu)	Northern Winter Wheat Zone	*Hap* L
40	Hongjinmai	China (Gansu)	Northern Winter Wheat Zone	*Hap* L
41	Lanhuamai	China (Gansu)	Northern Winter Wheat Zone	*Hap* H
42	Shanxibaimai	China (Gansu)	Northern Winter Wheat Zone	*Hap* L
43	Baiqiumai	China (Hebei)	Northern Winter Wheat Zone	*Hap* L
44	Hongmai	China (Hebei)	Northern Winter Wheat Zone	Unknown
45	Xiaobaimang	China (Hebei)	Northern Winter Wheat Zone	*Hap* L
46	Zhuoludongmai	China (Hebei)	Northern Winter Wheat Zone	*Hap* L
47	Mahuaban	China (Jilin)	Northern Winter Wheat Zone	*Hap* H
48	Panshiwumang	China (Jilin)	Northern Winter Wheat Zone	*Hap* H
49	Youmangbaifu	China (Jilin)	Northern Winter Wheat Zone	*Hap* H

50	Xiaokouhong	China (Liaoning)	Northern Winter Wheat Zone	*Hap* L
51	Youmangbaifu	China (Liaoning)	Northern Winter Wheat Zone	*Hap* H
52	Honglaomai	China (Shannxi)	Northern Winter Wheat Zone	*Hap* H
53	Laomai	China (Shannxi)	Northern Winter Wheat Zone	*Hap* L
54	Hongpidongmai	China (Shanxi)	Northern Winter Wheat Zone	*Hap* H
55	Jiahongmai	China (Shanxi)	Northern Winter Wheat Zone	*Hap* H
56	Niuzhijia	China (Shanxi)	Northern Winter Wheat Zone	*Hap* H
57	Daimanghongmai	China (Tianjin)	Northern Winter Wheat Zone	*Hap* L
58	Baidatou	China (Gansu)	Northwestern Spring Wheat Zone	*Hap* L
59	Baiqitou	China (Gansu)	Northwestern Spring Wheat Zone	*Hap* L
60	Dabaimai	China (Gansu)	Northwestern Spring Wheat Zone	*Hap* L
61	Hongmangmai	China (Gansu)	Northwestern Spring Wheat Zone	*Hap* L
62	Huangjinmai	China (Gansu)	Northwestern Spring Wheat Zone	*Hap* L
63	Huoliyan	China (Gansu)	Northwestern Spring Wheat Zone	*Hap* L
64	Yizhimai	China (Gansu)	Northwestern Spring Wheat Zone	*Hap* L
65	Baimazha	China (Ningxia)	Northwestern Spring Wheat Zone	*Hap* L
66	Hongtuzi	China (Ningxia)	Northwestern Spring Wheat Zone	*Hap* L
67	Laotutou	China (Ningxia)	Northwestern Spring Wheat Zone	*Hap* L
68	Shanmai	China (Ningxia)	Northwestern Spring Wheat Zone	*Hap* L
69	Shanmai	China (Ningxia)	Northwestern Spring Wheat Zone	*Hap* L
70	Dabaimai	China (Qinghai)	Northwestern Spring Wheat Zone	Unknown
71	Galaohan	China (Qinghai)	Northwestern Spring Wheat Zone	*Hap* L
72	Gejiaxiang	China (Qinghai)	Qinghai-Tibetan Plateau Spring-Winter Wheat Zone	*Hap* L
73	Liuyuehuang	China (Qinghai)	Qinghai-Tibetan Plateau Spring-Winter Wheat Zone	*Hap* L
74	Baimangxiaomai	China (Sichuan)	Qinghai-Tibetan Plateau Spring-Winter Wheat Zone	*Hap* H
75	Bendihuanghuamai	China (Sichuan)	Qinghai-Tibetan Plateau Spring-Winter Wheat Zone	*Hap* L

76	Dachunbaisilengmai	China (Sichuan)	Qinghai-Tibetan Plateau Spring-Winter Wheat Zone	*Hap* L
77	Kangdingxiaomai	China (Sichuan)	Qinghai-Tibetan Plateau Spring-Winter Wheat Zone	*Hap* L
78	Bailanghuimai	China (Xizang)	Qinghai-Tibetan Plateau Spring-Winter Wheat Zone	*Hap* L
79	Bianbachunmai 6	China (Xizang)	Qinghai-Tibetan Plateau Spring-Winter Wheat Zone	*Hap* L
80	Geerhongmai	China (Xizang)	Qinghai-Tibetan Plateau Spring-Winter Wheat Zone	*Hap* L
81	Motuoxiaomai	China (Xizang)	Qinghai-Tibetan Plateau Spring-Winter Wheat Zone	*Hap* L
82	Muzongzhuoga	China (Xizang)	Qinghai-Tibetan Plateau Spring-Winter Wheat Zone	*Hap* L
83	Wujiangzhuo	China (Xizang)	Qinghai-Tibetan Plateau Spring-Winter Wheat Zone	*Hap* L
84	Zhahong	China (Xizang)	Qinghai-Tibetan Plateau Spring-Winter Wheat Zone	*Hap* L
85	Shengen	China (Fujian)	Southern Autumn-Sown Spring Wheat Zone	*Hap* H
86	Songruimai	China (Fujian)	Southern Autumn-Sown Spring Wheat Zone	*Hap* H
87	Chaoanxiaomai	China (Guangdong)	Southern Autumn-Sown Spring Wheat Zone	*Hap* H
88	Chike	China (Guangdong)	Southern Autumn-Sown Spring Wheat Zone	*Hap* H
89	Shanglinxiaomai	China (Guangxi)	Southern Autumn-Sown Spring Wheat Zone	*Hap* H
90	Baihuamai	China (Guizhou)	Southwestern Autumn-Sown Spring Wheat Zone	*Hap* L
91	Baimangmai	China (Guizhou)	Southwestern Autumn-Sown Spring Wheat Zone	*Hap* H
92	Jiangmai	China (Guizhou)	Southwestern Autumn-Sown Spring Wheat Zone	*Hap* L
93	Yuqiumai	China (Guizhou)	Southwestern Autumn-Sown Spring Wheat Zone	*Hap* L
94	Hongmangzi	China (Hubei)	Southwestern Autumn-Sown Spring Wheat Zone	*Hap* L
95	Hanzhongbai	China (Shannxi)	Southwestern Autumn-Sown Spring Wheat Zone	*Hap* L
96	Hongxumai	China (Shannxi)	Southwestern Autumn-Sown Spring Wheat Zone	*Hap* L
97	Suotiaohongmai	China (Shannxi)	Southwestern Autumn-Sown Spring Wheat Zone	*Hap* L
98	Xiaosanyuehuang	China (Shannxi)	Southwestern Autumn-Sown Spring Wheat Zone	*Hap* L
99	Baimaizi	China (Sichuan)	Southwestern Autumn-Sown Spring Wheat Zone	*Hap* L
100	Chengduguangtou	China (Sichuan)	Southwestern Autumn-Sown Spring Wheat Zone	*Hap* L
101	Honghuamai	China (Sichuan)	Southwestern Autumn-Sown Spring Wheat Zone	*Hap* L

102	Huanxiangguo	China (Sichuan)	Southwestern Autumn-Sown Spring Wheat Zone	*Hap* L
103	Tongjiabaxiaomai	China (Sichuan)	Southwestern Autumn-Sown Spring Wheat Zone	*Hap* L
104	Yangmai	China (Sichuan)	Southwestern Autumn-Sown Spring Wheat Zone	*Hap* L
105	Zipi	China (Sichuan)	Southwestern Autumn-Sown Spring Wheat Zone	*Hap* H
106	Biantouguangkemai	China (Yunnan)	Southwestern Autumn-Sown Spring Wheat Zone	*Hap* L
107	Changmangshibiantou	China (Yunnan)	Southwestern Autumn-Sown Spring Wheat Zone	*Hap* L
108	Dianxihongkeyangmai	China (Yunnan)	Southwestern Autumn-Sown Spring Wheat Zone	*Hap* L
109	Yangmai	China (Yunnan)	Southwestern Autumn-Sown Spring Wheat Zone	*Hap* L
110	Zhugoumai	China (Yunnan)	Southwestern Autumn-Sown Spring Wheat Zone	*Hap* L
111	Zhushimai	China (Yunnan)	Southwestern Autumn-Sown Spring Wheat Zone	*Hap* L
112	Baidongmai	China (Xinjiang)	Xinjiang Winter-Spring Wheat Zone	*Hap* H
113	Chunmai	China (Xinjiang)	Xinjiang Winter-Spring Wheat Zone	*Hap* L
114	Hongchunmai	China (Xinjiang)	Xinjiang Winter-Spring Wheat Zone	*Hap* L
115	Hongchunmai	China (Xinjiang)	Xinjiang Winter-Spring Wheat Zone	*Hap* L
116	Hongdongmai	China (Xinjiang)	Xinjiang Winter-Spring Wheat Zone	*Hap* L
117	Hongdongmai	China (Xinjiang)	Xinjiang Winter-Spring Wheat Zone	*Hap* L
118	Hongdongmai	China (Xinjiang)	Xinjiang Winter-Spring Wheat Zone	*Hap* L
119	Hongjinbaoyin	China (Xinjiang)	Xinjiang Winter-Spring Wheat Zone	*Hap* L
120	Wumangchunmai	China (Xinjiang)	Xinjiang Winter-Spring Wheat Zone	*Hap* L
121	Wumangchunmai	China (Xinjiang)	Xinjiang Winter-Spring Wheat Zone	*Hap* L
122	Buyanghong	China (Anhui)	Yellow and Huai River Valleys Facultative Wheat Zone	Unknown
123	Baiqimai	China (Gansu)	Yellow and Huai River Valleys Facultative Wheat Zone	*Hap* L
124	Huomai	China (Gansu)	Yellow and Huai River Valleys Facultative Wheat Zone	*Hap* L
125	Baimangmai	China (Hebei)	Yellow and Huai River Valleys Facultative Wheat Zone	*Hap* L
126	Baitiaoyu	China (Henan)	Yellow and Huai River Valleys Facultative Wheat Zone	*Hap* H
127	Chushanbao	China (Henan)	Yellow and Huai River Valleys Facultative Wheat Zone	*Hap* L

128	Dakoumai	China (Henan)	Yellow and Huai River Valleys Facultative Wheat Zone	*Hap* L
129	Fumai	China (Henan)	Yellow and Huai River Valleys Facultative Wheat Zone	*Hap* L
130	Honggoudou	China (Henan)	Yellow and Huai River Valleys Facultative Wheat Zone	*Hap* L
131	Hongheshangtou	China (Henan)	Yellow and Huai River Valleys Facultative Wheat Zone	*Hap* H
132	Huangguaxian	China (Henan)	Yellow and Huai River Valleys Facultative Wheat Zone	*Hap* L
133	Jiangmai	China (Henan)	Yellow and Huai River Valleys Facultative Wheat Zone	*Hap* L
134	Pingyuan 50	China (Henan)	Yellow and Huai River Valleys Facultative Wheat Zone	*Hap* H
135	Sanyuehuang	China (Henan)	Yellow and Huai River Valleys Facultative Wheat Zone	*Hap* H
136	Youmangsaogudan	China (Henan)	Yellow and Huai River Valleys Facultative Wheat Zone	*Hap* L
137	Youzimai	China (Henan)	Yellow and Huai River Valleys Facultative Wheat Zone	*Hap* L
138	Dayuhua	China (Jiangsu)	Yellow and Huai River Valleys Facultative Wheat Zone	*Hap* H
139	Meiqianwu	China (Jiangsu)	Yellow and Huai River Valleys Facultative Wheat Zone	*Hap* H
140	Baibiansui	China (Shandong)	Yellow and Huai River Valleys Facultative Wheat Zone	*Hap* L
141	Baituzitou	China (Shandong)	Yellow and Huai River Valleys Facultative Wheat Zone	*Hap* L
142	Banjiemang	China (Shandong)	Yellow and Huai River Valleys Facultative Wheat Zone	*Hap* L
143	Dalibanmang	China (Shandong)	Yellow and Huai River Valleys Facultative Wheat Zone	*Hap* L
144	Laolaixia	China (Shandong)	Yellow and Huai River Valleys Facultative Wheat Zone	*Hap* H
145	Louguding	China (Shandong)	Yellow and Huai River Valleys Facultative Wheat Zone	*Hap* L
146	Xishanbiansui	China (Shandong)	Yellow and Huai River Valleys Facultative Wheat Zone	*Hap* L
147	Zijiehong	China (Shandong)	Yellow and Huai River Valleys Facultative Wheat Zone	Unknown
148	Baimangmai	China (Shannxi)	Yellow and Huai River Valleys Facultative Wheat Zone	*Hap* L
149	Hongqiangchang	China (Shannxi)	Yellow and Huai River Valleys Facultative Wheat Zone	*Hap* L
150	Laoqimai	China (Shannxi)	Yellow and Huai River Valleys Facultative Wheat Zone	*Hap* L
151	Mazhamai	China (Shannxi)	Yellow and Huai River Valleys Facultative Wheat Zone	*Hap* L
152	Qiangchangmai	China (Shannxi)	Yellow and Huai River Valleys Facultative Wheat Zone	*Hap* L

153	Tumangmai	China (Shannxi)	Yellow and Huai River Valleys Facultative Wheat Zone	*Hap* L
154	Baihuomai	China (Shanxi)	Yellow and Huai River Valleys Facultative Wheat Zone	*Hap* L
155	Sanyuehuang	Unknown	Yellow and Huai River Valleys Facultative Wheat Zone	*Hap* L
156	Xiaofoshou	Unknown	Yellow and Huai River Valleys Facultative Wheat Zone	*Hap* H
157	Zhongguochun	Unknown	Unknown	*Hap* L

Supplementary Table S5. Basic information of 348 accessions (names, origins, ecological zones and released dates) and their sequence polymorphism assays of *TaMOC1-7A*

**No.**	**Name**	**Origin**	**Ecological Zones**	**Decade**	***TaMOC1-7A* Haplotype**
1	Anhui 3	China (Anhui)	Middle and Lower Yangtze Valleys Autumn-Sown Spring Wheat Zone	Pre-1960	*Hap* L
2	Anhui 9	China (Anhui)	Middle and Lower Yangtze Valleys Autumn-Sown Spring Wheat Zone	Pre-1960	*Hap* L
3	Huadong 6	China (Jiangsu)	Middle and Lower Yangtze Valleys Autumn-Sown Spring Wheat Zone	Pre-1960	*Hap* L
4	Wangmai 17	China (Jiangsu)	Middle and Lower Yangtze Valleys Autumn-Sown Spring Wheat Zone	Pre-1960	*Hap* L
5	Huadong 10	China (Jiangsu)	Middle and Lower Yangtze Valleys Autumn-Sown Spring Wheat Zone	Pre-1960	*Hap* H
6	Liying 1	China (Jiangsu)	Middle and Lower Yangtze Valleys Autumn-Sown Spring Wheat Zone	Pre-1960	*Hap* H
7	Liying 5	China (Jiangsu)	Middle and Lower Yangtze Valleys Autumn-Sown Spring Wheat Zone	Pre-1960	*Hap* H
8	Jiulan	China (Zhejiang)	Middle and Lower Yangtze Valleys Autumn-Sown Spring Wheat Zone	Pre-1960	*Hap* H
9	Yimai 1	China (Hubei)	Middle and Lower Yangtze Valleys Autumn-Sown Spring Wheat Zone	Pre-1960	*Hap* H
10	Anhui 11	China (Anhui)	Middle and Lower Yangtze Valleys Autumn-Sown Spring Wheat Zone	1960s	*Hap* L

11	Mengfeng 8	China (Anhui)	Middle and Lower Yangtze Valleys Autumn-Sown Spring Wheat Zone	1960s	*Hap* L
12	Zhongshan 9	China (Jiangsu)	Middle and Lower Yangtze Valleys Autumn-Sown Spring Wheat Zone	1960s	*Hap* L
13	Emai 6	China (Hubei)	Middle and Lower Yangtze Valleys Autumn-Sown Spring Wheat Zone	1960s	*Hap* H
14	Jingzhou 2	China (Hubei)	Middle and Lower Yangtze Valleys Autumn-Sown Spring Wheat Zone	1960s	*Hap* L
15	Zhongjiwan	China (Hunan)	Middle and Lower Yangtze Valleys Autumn-Sown Spring Wheat Zone	1960s	*Hap* L
16	Wanyuan 28 Dali	China (Henan)	Middle and Lower Yangtze Valleys Autumn-Sown Spring Wheat Zone	1970s	*Hap* L
17	Siyang 117	China (Jiangsu)	Middle and Lower Yangtze Valleys Autumn-Sown Spring Wheat Zone	1970s	*Hap* L
18	Sumai 3	China (Jiangsu)	Middle and Lower Yangtze Valleys Autumn-Sown Spring Wheat Zone	1970s	*Hap* L
19	Sujian 14	China (Jiangsu)	Middle and Lower Yangtze Valleys Autumn-Sown Spring Wheat Zone	1970s	*Hap* L
20	Nannong 2293xuan	China (Jiangsu)	Middle and Lower Yangtze Valleys Autumn-Sown Spring Wheat Zone	1970s	*Hap* L
21	Zhemai 1	China (Zhejiang)	Middle and Lower Yangtze Valleys Autumn-Sown Spring Wheat Zone	1970s	*Hap* L
22	Jiamai 25	China (Zhejiang)	Middle and Lower Yangtze Valleys Autumn-Sown Spring Wheat Zone	1970s	*Hap* L
23	Nanzhongzao	China (Zhejiang)	Middle and Lower Yangtze Valleys Autumn-Sown Spring Wheat Zone	1970s	*Hap* L

24	Zhexuan 78-23	China (Zhejiang)	Middle and Lower Yangtze Valleys Autumn-Sown Spring Wheat Zone	1970s	*Hap* H
25	Xiangmai 8	China (Hubei)	Middle and Lower Yangtze Valleys Autumn-Sown Spring Wheat Zone	1970s	*Hap* L
26	Xiang 791-2	China (Hunan)	Middle and Lower Yangtze Valleys Autumn-Sown Spring Wheat Zone	1970s	*Hap* L
27	Zhongana 875	China (Hunan)	Middle and Lower Yangtze Valleys Autumn-Sown Spring Wheat Zone	1970s	*Hap* L
28	Wanpin 8203	China (Anhui)	Middle and Lower Yangtze Valleys Autumn-Sown Spring Wheat Zone	1980s	*Hap* L
29	Wan 85-50-Fan3	China (Anhui)	Middle and Lower Yangtze Valleys Autumn-Sown Spring Wheat Zone	1980s	*Hap* L
30	Wanpin 8337	China (Anhui)	Middle and Lower Yangtze Valleys Autumn-Sown Spring Wheat Zone	1980s	*Hap* L
31	Kang R16	China (Jiangsu)	Middle and Lower Yangtze Valleys Autumn-Sown Spring Wheat Zone	1980s	*Hap* L
32	Ning 8343	China (Jiangsu)	Middle and Lower Yangtze Valleys Autumn-Sown Spring Wheat Zone	1980s	*Hap* L
33	Ning 8537	China (Jiangsu)	Middle and Lower Yangtze Valleys Autumn-Sown Spring Wheat Zone	1980s	*Hap* H
34	Ningmaizi 3	China (Jiangsu)	Middle and Lower Yangtze Valleys Autumn-Sown Spring Wheat Zone	1980s	*Hap* L
35	Ningmaizi 13	China (Jiangsu)	Middle and Lower Yangtze Valleys Autumn-Sown Spring Wheat Zone	1980s	*Hap* L
36	Ning 8924	China (Jiangsu)	Middle and Lower Yangtze Valleys Autumn-Sown Spring Wheat Zone	1980s	*Hap* L

37	Ningmaizi 19	China (Jiangsu)	Middle and Lower Yangtze Valleys Autumn-Sown Spring Wheat Zone	1980s	*Hap* L
38	Nanda 8910	China (Jiangsu)	Middle and Lower Yangtze Valleys Autumn-Sown Spring Wheat Zone	1980s	*Hap* H
39	Limai 16	China (Zhejiang)	Middle and Lower Yangtze Valleys Autumn-Sown Spring Wheat Zone	1980s	*Hap* L
40	Zhemai 4	China (Zhejiang)	Middle and Lower Yangtze Valleys Autumn-Sown Spring Wheat Zone	1980s	*Hap* L
41	Exi 84-1031	China (Hubei)	Middle and Lower Yangtze Valleys Autumn-Sown Spring Wheat Zone	1980s	*Hap* L
42	Enmai 4	China (Hubei)	Middle and Lower Yangtze Valleys Autumn-Sown Spring Wheat Zone	1980s	*Hap* L
43	E 811	China (Hubei)	Middle and Lower Yangtze Valleys Autumn-Sown Spring Wheat Zone	1980s	*Hap* L
44	Xiangmai 12	China (Hunan)	Middle and Lower Yangtze Valleys Autumn-Sown Spring Wheat Zone	1980s	*Hap* L
45	Wan 89193	China (Anhui)	Middle and Lower Yangtze Valleys Autumn-Sown Spring Wheat Zone	1990s	*Hap* H
46	Wanpin 8410	China (Anhui)	Middle and Lower Yangtze Valleys Autumn-Sown Spring Wheat Zone	1990s	*Hap* H
47	Ningmaizi 44	China (Jiangsu)	Middle and Lower Yangtze Valleys Autumn-Sown Spring Wheat Zone	1990s	*Hap* L
48	Yangmai 158	China (Jiangsu)	Middle and Lower Yangtze Valleys Autumn-Sown Spring Wheat Zone	1990s	*Hap* L
49	Exi 652	China (Hubei)	Middle and Lower Yangtze Valleys Autumn-Sown Spring Wheat Zone	Unknown	*Hap* L

50	Yixi 102	China (Hubei)	Middle and Lower Yangtze Valleys Autumn-Sown Spring Wheat Zone	Unknown	*Hap* L
51	Gan 162	China (Jiangxi)	Middle and Lower Yangtze Valleys Autumn-Sown Spring Wheat Zone	Unknown	*Hap* H
52	Dongnong 101	China (Heilongjiang)	Northeastern Spring Wheat Zone	Pre-1960	*Hap* L
53	Hezuo 3	China (Heilongjiang)	Northeastern Spring Wheat Zone	Pre-1960	*Hap* L
54	Jia 6268A-549	China (Heilongjiang)	Northeastern Spring Wheat Zone	1960s	*Hap* L
55	Kequn	China (Heilongjiang)	Northeastern Spring Wheat Zone	1960s	*Hap* L
56	Xinshuguang 1	China (Heilongjiang)	Northeastern Spring Wheat Zone	1960s	*Hap* L
57	Xinshuguang 6	China (Heilongjiang)	Northeastern Spring Wheat Zone	1960s	*Hap* L
58	Hei 78-1259	China (Heilongjiang)	Northeastern Spring Wheat Zone	1970s	*Hap* L
59	Kefeng 3	China (Heilongjiang)	Northeastern Spring Wheat Zone	1970s	*Hap* L
60	Kelao 4	China (Heilongjiang)	Northeastern Spring Wheat Zone	1970s	*Hap* L
61	Kenbei 1	China (Heilongjiang)	Northeastern Spring Wheat Zone	1970s	*Hap* L
62	Kenda 1	China (Heilongjiang)	Northeastern Spring Wheat Zone	1970s	*Hap* L
63	Long 79-9468	China (Heilongjiang)	Northeastern Spring Wheat Zone	1970s	*Hap* L
64	Fengqiang 3	China (Jilin)	Northeastern Spring Wheat Zone	1970s	*Hap* L
65	Heifu 84S1378	China (Heilongjiang)	Northeastern Spring Wheat Zone	1980s	*Hap* H
66	Jia 84-S437	China (Heilongjiang)	Northeastern Spring Wheat Zone	1980s	*Hap* H
67	Longfumai 2	China (Heilongjiang)	Northeastern Spring Wheat Zone	1980s	*Hap* H
68	Longmai 18	China (Heilongjiang)	Northeastern Spring Wheat Zone	1980s	*Hap* L
69	Longmai 19	China (Heilongjiang)	Northeastern Spring Wheat Zone	1980s	*Hap* L
70	Xinkehan 9	China (Heilongjiang)	Northeastern Spring Wheat Zone	1980s	*Hap* H
71	Jichun 1016	China (Jilin)	Northeastern Spring Wheat Zone	1980s	*Hap* L
72	Kenhong 15	China (Heilongjiang)	Northeastern Spring Wheat Zone	1990s	*Hap* L
73	Xiaobingmai 33	China (Jilin)	Northeastern Spring Wheat Zone	1990s	*Hap* L

74	Lianglaiyoubaipixiaomai	China (Neimenggu)	Northern Spring Wheat Zone	Pre-1960	*Hap* L
75	Bihongsui	China (Neimenggu)	Northern Spring Wheat Zone	Pre-1960	*Hap* L
76	Jinghong 5	China (Beijing)	Northern Spring Wheat Zone	1960s	*Hap* L
77	Jiba 7529	China (Hebei)	Northern Spring Wheat Zone	1970s	*Hap* L
78	Yanbei 8	China (Shanxi)	Northern Spring Wheat Zone	1970s	*Hap* L
79	Jinchun 3	China (Shanxi)	Northern Spring Wheat Zone	1970s	*Hap* L
80	Neimai 11	China (Neimenggu)	Northern Spring Wheat Zone	1970s	*Hap* L
81	Pinchun 14	China (Beijing)	Northern Spring Wheat Zone	1980s	*Hap* L
82	Jichun 8055-1	China (Hebei)	Northern Spring Wheat Zone	1980s	*Hap* L
83	Dingxian 72	China (Beijing)	Northern Winter Wheat Zone	Pre-1960	*Hap* H
84	Huabei 672	China (Beijing)	Northern Winter Wheat Zone	Pre-1960	*Hap* L
85	Nongda 183	China (Beijing)	Northern Winter Wheat Zone	Pre-1960	*Hap* H
86	Henong 3	China (Hebei)	Northern Winter Wheat Zone	Pre-1960	*Hap* L
87	Mingxian 169	China (Shanxi)	Northern Winter Wheat Zone	Pre-1960	*Hap* H
88	Beijing 8	China (Beijing)	Northern Winter Wheat Zone	1960s	*Hap* L
89	Nongda 139	China (Beijing)	Northern Winter Wheat Zone	1960s	*Hap* L
90	Nongda 311	China (Beijing)	Northern Winter Wheat Zone	1960s	*Hap* L
91	Dongfanghong 3	China (Beijing)	Northern Winter Wheat Zone	1960s	*Hap* L
92	Keyi 23	China (Beijing)	Northern Winter Wheat Zone	1960s	*Hap* L
93	Taifu 1	China (Shanxi)	Northern Winter Wheat Zone	1960s	*Hap* H
94	Jinmai 8	China (Shanxi)	Northern Winter Wheat Zone	1960s	*Hap* L
95	Jinzhong 103	China (Shanxi)	Northern Winter Wheat Zone	1960s	*Hap* L
96	Gongnong 4	China (Shanxi)	Northern Winter Wheat Zone	1960s	*Hap* L
97	Pingliang 30	China (Gansu)	Northern Winter Wheat Zone	1960s	*Hap* L
98	Beijing 15	China (Beijing)	Northern Winter Wheat Zone	1970s	*Hap* L
99	Kecheng 1	China (Beijing)	Northern Winter Wheat Zone	1970s	*Hap* L

100	Fengkang 2	China (Beijing)	Northern Winter Wheat Zone	1970s	*Hap* L
101	Fengkang 8	China (Beijing)	Northern Winter Wheat Zone	1970s	*Hap* L
102	Jimai 17	China (Hebei)	Northern Winter Wheat Zone	1970s	*Hap* L
103	Tang 78042	China (Hebei)	Northern Winter Wheat Zone	1970s	*Hap* H
104	Jinmai 16	China (Shanxi)	Northern Winter Wheat Zone	1970s	*Hap* H
105	Lvhan 328	China (Shanxi)	Northern Winter Wheat Zone	1970s	*Hap* L
106	Jinmai 11	China (Shanxi)	Northern Winter Wheat Zone	1970s	*Hap* L
107	Taiyuan 2112	China (Shanxi)	Northern Winter Wheat Zone	1970s	*Hap* L
108	Yanan 11	China (Shaanxi)	Northern Winter Wheat Zone	1970s	*Hap* L
109	Yanan 18	China (Shaanxi)	Northern Winter Wheat Zone	1970s	*Hap* L
110	Pingliang 32	China (Gansu)	Northern Winter Wheat Zone	1970s	*Hap* L
111	Jingnong 81-49	China (Beijing)	Northern Winter Wheat Zone	1980s	*Hap* L
112	Jinghua 1	China (Beijing)	Northern Winter Wheat Zone	1980s	*Hap* L
113	Beinongda BL8	China (Beijing)	Northern Winter Wheat Zone	1980s	*Hap* L
114	Hebuyu 6068	China (Beijing)	Northern Winter Wheat Zone	1980s	*Hap* L
115	Jingnong 86-89	China (Beijing)	Northern Winter Wheat Zone	1980s	*Hap* L
116	Xiaoshan 8	China (Beijing)	Northern Winter Wheat Zone	1980s	*Hap* L
117	Jingpin 12	China (Beijing)	Northern Winter Wheat Zone	1980s	*Hap* H
118	Pinkang 244	China (Beijing)	Northern Winter Wheat Zone	1980s	*Hap* L
119	Yuandong 821	China (Beijing)	Northern Winter Wheat Zone	1980s	*Hap* L
120	Yuandong 822	China (Beijing)	Northern Winter Wheat Zone	1980s	*Hap* L
121	An 85 Zhong 124-1	China (Beijing)	Northern Winter Wheat Zone	1980s	*Hap* L
122	Qiaoliang BW41	China (Beijing)	Northern Winter Wheat Zone	1980s	*Hap* L
123	Zhongda 89-60192-2	China (Beijing)	Northern Winter Wheat Zone	1980s	*Hap* L
124	Beijing 8694	China (Beijing)	Northern Winter Wheat Zone	1980s	*Hap* L
125	Lang 8302	China (Hebei)	Northern Winter Wheat Zone	1980s	*Hap* L

126	Tang 85-5032	China (Hebei)	Northern Winter Wheat Zone	1980s	*Hap* L
127	Jinmai 31	China (Shanxi)	Northern Winter Wheat Zone	1980s	*Hap* L
128	Jin 3052-5	China (Shanxi)	Northern Winter Wheat Zone	1980s	*Hap* L
129	Changzhi 5557	China (Shanxi)	Northern Winter Wheat Zone	1980s	*Hap* L
130	Changzhi 6406	China (Shanxi)	Northern Winter Wheat Zone	1980s	*Hap* H
131	Xifeng 16	China (Gansu)	Northern Winter Wheat Zone	1980s	*Hap* H
132	Qingshui 15-41(2)	China (Gansu)	Northern Winter Wheat Zone	1980s	*Hap* L
133	Jingnong 94-32	China (Beijing)	Northern Winter Wheat Zone	1990s	*Hap* L
134	Jinghe 91-P19	China (Beijing)	Northern Winter Wheat Zone	1990s	*Hap* L
135	Pindong 904110-3	China (Beijing)	Northern Winter Wheat Zone	1990s	*Hap* L
136	Zhongyou 9507	China (Beijing)	Northern Winter Wheat Zone	1990s	*Hap* L
137	Taiyuan 351	China (Shanxi)	Northern Winter Wheat Zone	1990s	*Hap* H
138	Jin 1410	China (Shanxi)	Northern Winter Wheat Zone	Unknown	*Hap* L
139	Ganmai 6	China (Gansu)	Northwestern Spring Wheat Zone	Pre-1960	*Hap* L
140	Ganmai 8	China (Gansu)	Northwestern Spring Wheat Zone	1960s	*Hap* L
141	Dingxi 24	China (Gansu)	Northwestern Spring Wheat Zone	1960s	*Hap* L
142	Ganmai 46	China (Gansu)	Northwestern Spring Wheat Zone	1960s	*Hap* L
143	Hongtu	China (Ningxia)	Northwestern Spring Wheat Zone	1960s	*Hap* L
144	Xiangnong 3	China (Qinghai)	Northwestern Spring Wheat Zone	1960s	*Hap* L
145	Huzhuhong	China (Qinghai)	Northwestern Spring Wheat Zone	1960s	*Hap* L
146	Longchun 7	China (Gansu)	Northwestern Spring Wheat Zone	1970s	*Hap* L
147	Linnong 12	China (Gansu)	Northwestern Spring Wheat Zone	1970s	*Hap* L
148	Jinmai 4	China (Gansu)	Northwestern Spring Wheat Zone	1970s	*Hap* L
149	Wudu 5	China (Gansu)	Northwestern Spring Wheat Zone	1970s	*Hap* L
150	Xifeng 9	China (Gansu)	Northwestern Spring Wheat Zone	1970s	*Hap* L
151	Xifeng 10	China (Gansu)	Northwestern Spring Wheat Zone	1970s	*Hap* L

152	Qingfeng 1	China (Gansu)	Northwestern Spring Wheat Zone	1970s	*Hap* L
153	Zhangchun 9	China (Gansu)	Northwestern Spring Wheat Zone	1970s	*Hap* L
154	Huining 5	China (Gansu)	Northwestern Spring Wheat Zone	1970s	*Hap* L
155	Huining 10	China (Gansu)	Northwestern Spring Wheat Zone	1970s	*Hap* L
156	Wuchun 1	China (Gansu)	Northwestern Spring Wheat Zone	1970s	*Hap* L
157	Jinmai 303	China (Gansu)	Northwestern Spring Wheat Zone	1970s	*Hap* L
158	Ningchun 4	China (Ningxia)	Northwestern Spring Wheat Zone	1970s	*Hap* L
159	Gaoyuan 506	China (Qinghai)	Northwestern Spring Wheat Zone	1970s	*Hap* L
160	Qingchun 25	China (Qinghai)	Northwestern Spring Wheat Zone	1970s	*Hap* L
161	Qingchun 28	China (Qinghai)	Northwestern Spring Wheat Zone	1970s	*Hap* L
162	Gaoyuan 338	China (Qinghai)	Northwestern Spring Wheat Zone	1970s	*Hap* L
163	Gan Tal21-10-2	China (Gansu)	Northwestern Spring Wheat Zone	1980s	*Hap* L
164	Gan 8221-1-1	China (Gansu)	Northwestern Spring Wheat Zone	1980s	*Hap* L
165	Gan 8358-2	China (Gansu)	Northwestern Spring Wheat Zone	1980s	*Hap* L
166	Shi 886	China (Ningxia)	Northwestern Spring Wheat Zone	1980s	*Hap* L
167	Ning 87N2801	China (Ningxia)	Northwestern Spring Wheat Zone	1980s	*Hap* L
168	Gaoyuan 602	China (Qinghai)	Northwestern Spring Wheat Zone	1980s	*Hap* L
169	Longdong 1	China (Gansu)	Northwestern Spring Wheat Zone	1990s	*Hap* L
170	Jian 72	China (Ningxia)	Northwestern Spring Wheat Zone	Unknown	*Hap* L
171	Rikaze 7	China (Xizang)	Qinghai-Tibetan Plateau Spring-Winter Wheat Zone	1960s	*Hap* L
172	Rikaze 8	China (Xizang)	Qinghai-Tibetan Plateau Spring-Winter Wheat Zone	1960s	*Hap* L
173	Zangdong 4	China (Xizang)	Qinghai-Tibetan Plateau Spring-Winter Wheat Zone	1970s	*Hap* L

174	Rikaze 54	China (Xizang)	Qinghai-Tibetan Plateau Spring-Winter Wheat Zone	1970s	*Hap* L
175	Taizhong 23	China (Taiwan)	Southern Autumn-Sown Spring Wheat Zone	Pre-1960	*Hap* L
176	Kangxiu 10	China (Fujian)	Southern Autumn-Sown Spring Wheat Zone	1960s	*Hap* L
177	Dixiuzao	China (Fujian)	Southern Autumn-Sown Spring Wheat Zone	1960s	*Hap* L
178	Yuanshan	China (Guangdong)	Southern Autumn-Sown Spring Wheat Zone	1960s	*Hap* L
179	Jinmai 2148	China (Fujian)	Southern Autumn-Sown Spring Wheat Zone	1970s	*Hap* L
180	Longxi 35	China (Fujian)	Southern Autumn-Sown Spring Wheat Zone	1970s	*Hap* H
181	Fufan 904	China (Fujian)	Southern Autumn-Sown Spring Wheat Zone	1980s	*Hap* L
182	Taizhongxuan 2	China (Taiwan)	Southern Autumn-Sown Spring Wheat Zone	1980s	*Hap* L
183	Hechang 45	China (Sichuan)	Southwestern Autumn-Sown Spring Wheat Zone	Pre-1960	*Hap* L
184	Shuwan 8	China (Sichuan)	Southwestern Autumn-Sown Spring Wheat Zone	Pre-1960	*Hap* L
185	Sichuan 51	China (Sichuan)	Southwestern Autumn-Sown Spring Wheat Zone	Pre-1960	*Hap* L
186	Chuanmai 10	China (Sichuan)	Southwestern Autumn-Sown Spring Wheat Zone	1960s	*Hap* L

187	Yaanzao	China (Sichuan)	Southwestern Autumn-Sown Spring Wheat Zone	1960s	*Hap* L
188	Fan 6	China (Sichuan)	Southwestern Autumn-Sown Spring Wheat Zone	1960s	*Hap* L
189	Xichangfanxiumai	China (Sichuan)	Southwestern Autumn-Sown Spring Wheat Zone	1960s	*Hap* L
190	Fengmai 11	China (Yunnan)	Southwestern Autumn-Sown Spring Wheat Zone	1960s	*Hap* L
191	Chuanmai 19	China (Sichuan)	Southwestern Autumn-Sown Spring Wheat Zone	1970s	*Hap* L
192	Hongai 1	China (Sichuan)	Southwestern Autumn-Sown Spring Wheat Zone	1970s	*Hap* L
193	Mianyang 11	China (Sichuan)	Southwestern Autumn-Sown Spring Wheat Zone	1970s	*Hap* L
194	Chuan 7911	China (Sichuan)	Southwestern Autumn-Sown Spring Wheat Zone	1970s	*Hap* L
195	Xichang 5548-9	China (Sichuan)	Southwestern Autumn-Sown Spring Wheat Zone	1970s	*Hap* L
196	Bimai 26	China (Guizhou)	Southwestern Autumn-Sown Spring Wheat Zone	1970s	*Hap* L
197	Yunmai 28	China (Yunnan)	Southwestern Autumn-Sown Spring Wheat Zone	1970s	*Hap* L
198	Yunmai 29	China (Yunnan)	Southwestern Autumn-Sown Spring Wheat Zone	1970s	*Hap* L
199	Nanyuan 1	China (Yunnan)	Southwestern Autumn-Sown Spring Wheat Zone	1970s	*Hap* L

200	Pu 170	China (Yunnan)	Southwestern Autumn-Sown Spring Wheat Zone	1970s	*Hap* L
201	Chuanmai 22	China (Sichuan)	Southwestern Autumn-Sown Spring Wheat Zone	1980s	*Hap* L
202	Chuanyu 12	China (Sichuan)	Southwestern Autumn-Sown Spring Wheat Zone	1980s	*Hap* L
203	Chuan 83C-1001	China (Sichuan)	Southwestern Autumn-Sown Spring Wheat Zone	1980s	*Hap* L
204	Pan 86001-3	China (Guizhou)	Southwestern Autumn-Sown Spring Wheat Zone	1980s	*Hap* L
205	Pan 88080-3-1-1	China (Guizhou)	Southwestern Autumn-Sown Spring Wheat Zone	1980s	*Hap* L
206	Bimai 10	China (Guizhou)	Southwestern Autumn-Sown Spring Wheat Zone	1980s	*Hap* L
207	Bimai 13	China (Guizhou)	Southwestern Autumn-Sown Spring Wheat Zone	1980s	*Hap* L
208	Guinong 10	China (Guizhou)	Southwestern Autumn-Sown Spring Wheat Zone	1980s	*Hap* L
209	Kenguia 1	China (Guizhou)	Southwestern Autumn-Sown Spring Wheat Zone	1980s	*Hap* L
210	Hemai 8052	China (Guizhou)	Southwestern Autumn-Sown Spring Wheat Zone	1980s	*Hap* L
211	Yunmai 33	China (Yunnan)	Southwestern Autumn-Sown Spring Wheat Zone	1980s	*Hap* L
212	Yunmai 34	China (Yunnan)	Southwestern Autumn-Sown Spring Wheat Zone	1980s	*Hap* L

213	Jingmai 2	China (Yunnan)	Southwestern Autumn-Sown Spring Wheat Zone	1980s	*Hap* L
214	Dian 8613	China (Yunnan)	Southwestern Autumn-Sown Spring Wheat Zone	1980s	*Hap* L
215	Mianyang 26	China (Sichuan)	Southwestern Autumn-Sown Spring Wheat Zone	1990s	*Hap* L
216	Xingzhuai 3	China (Guizhou)	Southwestern Autumn-Sown Spring Wheat Zone	1990s	*Hap* L
217	Guinong Y13	China (Guizhou)	Southwestern Autumn-Sown Spring Wheat Zone	1990s	*Hap* L
218	Gui 775	China (Guizhou)	Southwestern Autumn-Sown Spring Wheat Zone	1990s	*Hap* L
219	Xingyi 4	China (Guizhou)	Southwestern Autumn-Sown Spring Wheat Zone	1990s	*Hap* H
220	Qianjian 28	China (Guizhou)	Southwestern Autumn-Sown Spring Wheat Zone	Unknown	*Hap* L
221	Xingyingwu 3	China (Guizhou)	Southwestern Autumn-Sown Spring Wheat Zone	Unknown	*Hap* L
222	Dian 622-525-2	China (Yunnan)	Southwestern Autumn-Sown Spring Wheat Zone	Unknown	*Hap* H
223	Kashibaipi	China (Xinjiang)	Xinjiang Winter-Spring Wheat Zone	Pre-1960	*Hap* L
224	Xindong 2	China (Xinjiang)	Xinjiang Winter-Spring Wheat Zone	1960s	*Hap* H
225	Tuokexun 1	China (Xinjiang)	Xinjiang Winter-Spring Wheat Zone	1960s	*Hap* L
226	Kashi 1	China (Xinjiang)	Xinjiang Winter-Spring Wheat Zone	1970s	*Hap* L
227	Yinong 8	China (Xinjiang)	Xinjiang Winter-Spring Wheat Zone	1970s	*Hap* L
228	Changdong 5	China (Xinjiang)	Xinjiang Winter-Spring Wheat Zone	1980s	*Hap* L

229	Tuchun 6	China (Xinjiang)	Xinjiang Winter-Spring Wheat Zone	1980s	*Hap* L
230	Xinchun 2	China (Xinjiang)	Xinjiang Winter-Spring Wheat Zone	1980s	*Hap* L
231	Jiudong 2	China (Xinjiang)	Xinjiang Winter-Spring Wheat Zone	1980s	*Hap* L
232	Shite 14	China (Hebei)	Yellow and Huai River Valleys Facultative Wheat Zone	Pre-1960	*Hap* L
233	Shijiazhuang 407	China (Hebei)	Yellow and Huai River Valleys Facultative Wheat Zone	Pre-1960	*Hap* L
234	Shijiazhuang 4	China (Hebei)	Yellow and Huai River Valleys Facultative Wheat Zone	Pre-1960	*Hap* L
235	1817	China (Shanxi)	Yellow and Huai River Valleys Facultative Wheat Zone	Pre-1960	*Hap* H
236	Qida 195	China (Shandong)	Yellow and Huai River Valleys Facultative Wheat Zone	Pre-1960	*Hap* H
237	Jinan 2	China (Shandong)	Yellow and Huai River Valleys Facultative Wheat Zone	Pre-1960	*Hap* L
238	Neixiang 5	China (Henan)	Yellow and Huai River Valleys Facultative Wheat Zone	Pre-1960	*Hap* L
239	Jingyang 60	China (Shaanxi)	Yellow and Huai River Valleys Facultative Wheat Zone	Pre-1960	*Hap* L
240	Bima 1	China (Shaanxi)	Yellow and Huai River Valleys Facultative Wheat Zone	Pre-1960	*Hap* L
241	Bima 4	China (Shaanxi)	Yellow and Huai River Valleys Facultative Wheat Zone	Pre-1960	*Hap* L
242	Shannong 9	China (Shaanxi)	Yellow and Huai River Valleys Facultative Wheat Zone	Pre-1960	*Hap* L

243	Xinong 6028	China (Shaanxi)	Yellow and Huai River Valleys Facultative Wheat Zone	Pre-1960	*Hap* L
244	Jingyang 30	China (Shaanxi)	Yellow and Huai River Valleys Facultative Wheat Zone	Pre-1960	*Hap* L
245	Shijiazhuang 34	China (Hebei)	Yellow and Huai River Valleys Facultative Wheat Zone	1960s	*Hap* L
246	Shijiazhuang 54	China (Hebei)	Yellow and Huai River Valleys Facultative Wheat Zone	1960s	*Hap* L
247	Zaoxinshi	China (Hebei)	Yellow and Huai River Valleys Facultative Wheat Zone	1960s	*Hap* L
248	Hengshui 7004	China (Hebei)	Yellow and Huai River Valleys Facultative Wheat Zone	1960s	*Hap* L
249	Jinan 9	China (Shandong)	Yellow and Huai River Valleys Facultative Wheat Zone	1960s	*Hap* L
250	Youbao	China (Shandong)	Yellow and Huai River Valleys Facultative Wheat Zone	1960s	*Hap* L
251	Changwei 20	China (Shandong)	Yellow and Huai River Valleys Facultative Wheat Zone	1960s	*Hap* H
252	Yannong 3	China (Shandong)	Yellow and Huai River Valleys Facultative Wheat Zone	1960s	*Hap* L
253	Anxuan 2	China (Henan)	Yellow and Huai River Valleys Facultative Wheat Zone	1960s	*Hap* L
254	Zhengzhou 6	China (Henan)	Yellow and Huai River Valleys Facultative Wheat Zone	1960s	*Hap* L
255	Yuanzhu 55	China (Henan)	Yellow and Huai River Valleys Facultative Wheat Zone	1960s	*Hap* L

256	Zhengzhou 4	China (Henan)	Yellow and Huai River Valleys Facultative Wheat Zone	1960s	*Hap* L
257	Fengchan 3	China (Shaanxi)	Yellow and Huai River Valleys Facultative Wheat Zone	1960s	*Hap* H
258	Jinguangmai	China (Shaanxi)	Yellow and Huai River Valleys Facultative Wheat Zone	1960s	*Hap* H
259	Fuzhuang 30	China (Shaanxi)	Yellow and Huai River Valleys Facultative Wheat Zone	1960s	*Hap* L
260	Xiannong 39	China (Shaanxi)	Yellow and Huai River Valleys Facultative Wheat Zone	1960s	*Hap* L
261	Pin 39	China (Hebei)	Yellow and Huai River Valleys Facultative Wheat Zone	1970s	*Hap* L
262	Cangzhou 1	China (Hebei)	Yellow and Huai River Valleys Facultative Wheat Zone	1970s	*Hap* L
263	12040	China (Hebei)	Yellow and Huai River Valleys Facultative Wheat Zone	1970s	*Hap* L
264	Hengdalihong	China (Hebei)	Yellow and Huai River Valleys Facultative Wheat Zone	1970s	*Hap* L
265	Jimai 20	China (Hebei)	Yellow and Huai River Valleys Facultative Wheat Zone	1970s	*Hap* L
266	Jimai 14	China (Hebei)	Yellow and Huai River Valleys Facultative Wheat Zone	1970s	*Hap* L
267	Jinmai 20	China (Shanxi)	Yellow and Huai River Valleys Facultative Wheat Zone	1970s	*Hap* L
268	Taishan 1	China (Shandong)	Yellow and Huai River Valleys Facultative Wheat Zone	1970s	*Hap* L

269	Taishan 4	China (Shandong)	Yellow and Huai River Valleys Facultative Wheat Zone	1970s	*Hap* L
270	Lainongpinxi 22	China (Shandong)	Yellow and Huai River Valleys Facultative Wheat Zone	1970s	*Hap* L
271	Lumai 1	China (Shandong)	Yellow and Huai River Valleys Facultative Wheat Zone	1970s	*Hap* L
272	Lunong 784081	China (Shandong)	Yellow and Huai River Valleys Facultative Wheat Zone	1970s	*Hap* H
273	Taishan 7	China (Shandong)	Yellow and Huai River Valleys Facultative Wheat Zone	1970s	*Hap* H
274	Yannong 15	China (Shandong)	Yellow and Huai River Valleys Facultative Wheat Zone	1970s	*Hap* L
275	Bonong 7023	China (Henan)	Yellow and Huai River Valleys Facultative Wheat Zone	1970s	*Hap* L
276	Mengxian 2	China (Henan)	Yellow and Huai River Valleys Facultative Wheat Zone	1970s	*Hap* L
277	Wan 7107	China (Henan)	Yellow and Huai River Valleys Facultative Wheat Zone	1970s	*Hap* L
278	Bainong 3217	China (Henan)	Yellow and Huai River Valleys Facultative Wheat Zone	1970s	*Hap* L
279	Yumai 2	China (Henan)	Yellow and Huai River Valleys Facultative Wheat Zone	1970s	*Hap* L
280	Yu 7106-0-22-1-3-2B	China (Henan)	Yellow and Huai River Valleys Facultative Wheat Zone	1970s	*Hap* L
281	Zhengzhou 741	China (Henan)	Yellow and Huai River Valleys Facultative Wheat Zone	1970s	*Hap* L

282	Aifeng 3	China (Shaanxi)	Yellow and Huai River Valleys Facultative Wheat Zone	1970s	*Hap* H
283	Xiaoyan 4	China (Shaanxi)	Yellow and Huai River Valleys Facultative Wheat Zone	1970s	*Hap* L
284	Baomai 3	China (Shaanxi)	Yellow and Huai River Valleys Facultative Wheat Zone	1970s	*Hap* L
285	Baomai 5	China (Shaanxi)	Yellow and Huai River Valleys Facultative Wheat Zone	1970s	*Hap* L
286	Shan 70-1	China (Shaanxi)	Yellow and Huai River Valleys Facultative Wheat Zone	1970s	*Hap* H
287	Xiannong 151	China (Shaanxi)	Yellow and Huai River Valleys Facultative Wheat Zone	1970s	*Hap* L
288	Shan 7219-8-11-2-1	China (Shaanxi)	Yellow and Huai River Valleys Facultative Wheat Zone	1970s	*Hap* L
289	Xiaoyan 6	China (Shaanxi)	Yellow and Huai River Valleys Facultative Wheat Zone	1970s	*Hap* L
290	Ningfengmai	China (Jiangsu)	Yellow and Huai River Valleys Facultative Wheat Zone	1970s	*Hap* L
291	Huaimai 11	China (Jiangsu)	Yellow and Huai River Valleys Facultative Wheat Zone	1970s	*Hap* H
292	Xiaonong 76189	China (Anhui)	Yellow and Huai River Valleys Facultative Wheat Zone	1970s	*Hap* L
293	Zhangdong 29	China (Gansu)	Yellow and Huai River Valleys Facultative Wheat Zone	1970s	*Hap* L
294	Jimai 23	China (Hebei)	Yellow and Huai River Valleys Facultative Wheat Zone	1980s	*Hap* L

295	Shi 82-5201	China (Hebei)	Yellow and Huai River Valleys Facultative Wheat Zone	1980s	*Hap* H
296	Jimai 19	China (Hebei)	Yellow and Huai River Valleys Facultative Wheat Zone	1980s	*Hap* L
297	Jishi 5032	China (Hebei)	Yellow and Huai River Valleys Facultative Wheat Zone	1980s	*Hap* L
298	Jin 865096	China (Shanxi)	Yellow and Huai River Valleys Facultative Wheat Zone	1980s	*Hap* L
299	Jinmai 37	China (Shanxi)	Yellow and Huai River Valleys Facultative Wheat Zone	1980s	*Hap* L
300	Linyuan 129	China (Shanxi)	Yellow and Huai River Valleys Facultative Wheat Zone	1980s	*Hap* L
301	Pingyang 27	China (Shanxi)	Yellow and Huai River Valleys Facultative Wheat Zone	1980s	*Hap* L
302	Lumai 10	China (Shandong)	Yellow and Huai River Valleys Facultative Wheat Zone	1980s	*Hap* L
303	Lumai 5	China (Shandong)	Yellow and Huai River Valleys Facultative Wheat Zone	1980s	*Hap* H
304	Shannong PH85-4	China (Shandong)	Yellow and Huai River Valleys Facultative Wheat Zone	1980s	*Hap* L
305	Lunong 86(5)174	China (Shandong)	Yellow and Huai River Valleys Facultative Wheat Zone	1980s	*Hap* L
306	Luzi 0863169	China (Shandong)	Yellow and Huai River Valleys Facultative Wheat Zone	1980s	*Hap* L
307	Luzi 0884142	China (Shandong)	Yellow and Huai River Valleys Facultative Wheat Zone	1980s	*Hap* L

308	Teng 80-1-2	China (Shandong)	Yellow and Huai River Valleys Facultative Wheat Zone	1980s	*Hap* L
309	Yanzhong 144	China (Shandong)	Yellow and Huai River Valleys Facultative Wheat Zone	1980s	*Hap* L
310	Lumai 7	China (Shandong)	Yellow and Huai River Valleys Facultative Wheat Zone	1980s	*Hap* L
311	Lumai 9	China (Shandong)	Yellow and Huai River Valleys Facultative Wheat Zone	1980s	*Hap* L
312	Yumai 18	China (Henan)	Yellow and Huai River Valleys Facultative Wheat Zone	1980s	*Hap* L
313	Yumai 14	China (Henan)	Yellow and Huai River Valleys Facultative Wheat Zone	1980s	*Hap* L
314	Hua 852895-2	China (Henan)	Yellow and Huai River Valleys Facultative Wheat Zone	1980s	*Hap* L
315	Yu 30691-1-3	China (Henan)	Yellow and Huai River Valleys Facultative Wheat Zone	1980s	*Hap* L
316	Yumai 7	China (Henan)	Yellow and Huai River Valleys Facultative Wheat Zone	1980s	*Hap* L
317	Qinmai 8	China (Shaanxi)	Yellow and Huai River Valleys Facultative Wheat Zone	1980s	*Hap* H
318	Shannong 7859	China (Shaanxi)	Yellow and Huai River Valleys Facultative Wheat Zone	1980s	*Hap* L
319	Qinmai 3	China (Shaanxi)	Yellow and Huai River Valleys Facultative Wheat Zone	1980s	*Hap* L
320	Huaimai 12	China (Jiangsu)	Yellow and Huai River Valleys Facultative Wheat Zone	1980s	*Hap* L

321	Xuzhou 19	China (Jiangsu)	Yellow and Huai River Valleys Facultative Wheat Zone	1980s	*Hap* L
322	Xuzhou 21	China (Jiangsu)	Yellow and Huai River Valleys Facultative Wheat Zone	1980s	*Hap* L
323	Bu 84111	China (Anhui)	Yellow and Huai River Valleys Facultative Wheat Zone	1980s	*Hap* L
324	Wo 80	China (Anhui)	Yellow and Huai River Valleys Facultative Wheat Zone	1980s	*Hap* L
325	Pingliang 35	China (Gansu)	Yellow and Huai River Valleys Facultative Wheat Zone	1980s	*Hap* L
326	Ji 93C6 156-2	China (Hebei)	Yellow and Huai River Valleys Facultative Wheat Zone	1990s	*Hap* L
327	Han 4564	China (Hebei)	Yellow and Huai River Valleys Facultative Wheat Zone	1990s	*Hap* L
328	Jinan 17	China (Shandong)	Yellow and Huai River Valleys Facultative Wheat Zone	1990s	*Hap* L
329	Lumai 22	China (Shandong)	Yellow and Huai River Valleys Facultative Wheat Zone	1990s	*Hap* L
330	Laizhou 953	China (Shandong)	Yellow and Huai River Valleys Facultative Wheat Zone	1990s	*Hap* L
331	Yumai 54	China (Henan)	Yellow and Huai River Valleys Facultative Wheat Zone	1990s	*Hap* L
332	Lankao 906	China (Henan)	Yellow and Huai River Valleys Facultative Wheat Zone	1990s	*Hap* L
333	Zhengzhou 8761	China (Henan)	Yellow and Huai River Valleys Facultative Wheat Zone	1990s	*Hap* L

334	Zheng 87305-0-13	China (Henan)	Yellow and Huai River Valleys Facultative Wheat Zone	1990s	*Hap* H
335	Zhengzi R84019-0-7-4-0-1	China (Henan)	Yellow and Huai River Valleys Facultative Wheat Zone	1990s	*Hap* L
336	Wenmai 6	China (Henan)	Yellow and Huai River Valleys Facultative Wheat Zone	1990s	*Hap* L
337	Anmai 95 Zhong 35	China (Henan)	Yellow and Huai River Valleys Facultative Wheat Zone	1990s	*Hap* L
338	Liken 2	China (Shaanxi)	Yellow and Huai River Valleys Facultative Wheat Zone	1990s	*Hap* L
339	Shannong 229	China (Shaanxi)	Yellow and Huai River Valleys Facultative Wheat Zone	1990s	*Hap* L
340	Fen 22	China (Shaanxi)	Yellow and Huai River Valleys Facultative Wheat Zone	1990s	*Hap* H
341	Gaoyou 503	China (Shaanxi)	Yellow and Huai River Valleys Facultative Wheat Zone	1990s	*Hap* L
342	Xuzhou 22	China (Jiangsu)	Yellow and Huai River Valleys Facultative Wheat Zone	1990s	*Hap* L
343	Gaobi-13	China (Hebei)	Yellow and Huai River Valleys Facultative Wheat Zone	Unknown	*Hap* H
344	Maijin 1	China (Shanxi)	Yellow and Huai River Valleys Facultative Wheat Zone	Unknown	*Hap* H
345	Jinyang 1045	China (Shanxi)	Yellow and Huai River Valleys Facultative Wheat Zone	Unknown	*Hap* L
346	Huangtai 103	China (Shandong)	Yellow and Huai River Valleys Facultative Wheat Zone	Unknown	*Hap* L

347	Jimai 19	China (Shandong)	Yellow and Huai River Valleys Facultative Wheat Zone	Unknown	*Hap* L
348	Shan 8786-0-3	China (Shaanxi)	Yellow and Huai River Valleys Facultative Wheat Zone	Unknown	*Hap* H
